# High Resolution Infrared Spectroscopy in Support of Ozone Atmospheric Monitoring and Validation of the Potential Energy Function

**DOI:** 10.3390/molecules27030911

**Published:** 2022-01-28

**Authors:** Alain Barbe, Semen Mikhailenko, Evgeniya Starikova, Vladimir Tyuterev

**Affiliations:** 1 Groupe de Spectrométrie Moléculaire et Atmosphérique, UMR CNRS 7331, Université de Reims, UFR Sciences Exactes et Naturelles, CEDEX02, BP 1039-51687 Reims, France; Vladimir.ty@gmail.com; 2 Laboratory of Theoretical Spectroscopy, V.E. Zuev Institute of Atmospheric Optics SB RAS, 634055 Tomsk, Russia; semen@iao.ru (S.M.); starikova_e@iao.ru (E.S.); 3 Climate and Environmental Physics Laboratory, Ural Federal University, 19, Mira av., 620002 Yekaterinburg, Russia; 4 Laboratory of Quantum Mechanics of Molecules and Radiative Processes, Tomsk State University, 634050 Tomsk, Russia

**Keywords:** ozone molecule, infrared high-resolution spectroscopy, atmospheric applications, spectra of the 18 existing isotopic species, line intensities, potential energy function

## Abstract

The first part of this review is a brief reminder of general information concerning atmospheric ozone, particularly related to its formation, destruction, observations of its decrease in the stratosphere, and its increase in the troposphere as a result of anthropogenic actions and solutions. A few words are said about the abandonment of the Airbus project Alliance, which was expected to be the substitute of the supersonic Concorde. This project is over due to the theoretical evaluation of the impact of a fleet in the stratosphere and has been replaced by the A380, which is now operating. The largest part is devoted to calculations and observations of the transitions in the infrared range and their applications for the atmosphere based both on effective models (Hamiltonian, symmetry rules, and dipole moments) and ab initio calculations. The complementarities of the two approaches are clearly demonstrated, particularly for the creation of an exhaustive line list consisting of more than 300,000 lines reaching experimental accuracies (from 0.00004 to 0.001 cm^−1^) for positions and a sub percent for the intensities in the 10 microns region. This contributes to definitively resolving the issue of the observed discrepancies between line intensity data in different spectral regions: between the infrared and ultraviolet ranges, on the one hand, and between 10 and 5 microns on the other hand. The following section is devoted to the application of recent work to improve the knowledge about the behavior of potential function at high energies. A controversial issue related to the shape of the potential function in the transition state range near the dissociation is discussed.

## 1. Introduction

The ozone (O_3_) molecule is one of the well-known molecules, due particularly to the “ozone hole” discovered in the 1970s in Antarctica, which undoubtedly proved anthropogenic impact on the ozone layer [1,2]. This human impact has also been manifested in an increase of CO_2_ content in the atmosphere. Ozone decrease in the stratosphere has been observed by satellites [3,4,5,6,7,8,9] as well as by ground-based spectrometers [10]. The reason for this decrease has clearly been identified [1], allowing for a consensus leading to the Montreal protocol, which allows for the reduction of chlorofluorocarbon (CFC) emissions and, in consequence, their stratospheric decrease, and permits the recovery of correct levels around 2050. Note that this necessary reduction in human activity will be much more difficult in the case of CO_2_ emissions.

But ozone is also a very interesting molecule in other aspects, such as the observation of anomalous isotopic enrichment [11,12,13,14,15,16], or the fact that this molecule, having three identical atoms, was experimentally identified as an asymmetric top having three different rotational constants in the electronic ground state. This explains a very large number of devoted studies. Another feature is its capacity to absorb radiation in a large wavelength range, that is microwave, far and medium infrared (IR), and visible and ultraviolet (UV) ranges, permitting atmospheric observations in all these domains. In order to accurately quantify the ozone concentration (no worse than to 1%), this requires a precise spectroscopy in all spectral ranges, explaining the very large number of spectroscopic studies, both experimental and theoretical. Two examples of problems arise. The first is the difference in the total ozone column retrieved using two different spectral ranges (10 and 5 microns) in infrared, and the second is between the IR and UV regions, this last one amounting to about 4%. The main part of this article, as indicated in the abstract, is devoted to high resolution infrared spectroscopy, which has a direct use in atmospheric applications. Three of these direct applications are particularly highlighted: the problem of agreement with an accuracy of 1% between spectroscopic data using suitable line lists in the infrared range has been solved [17,18,19,20,21,22,23,24]; the proof of the absence of a submerged barrier in the transition state range of the potential energy surface, having an impact on the modeling of isotopic exchange reactions in the O + OO collisions [25,26,27,28,29,30,31], which in turn could contribute to the anomalous isotope effect in ozone formation [11,12,13,14,15,16]; and a possible impact of the interactions among potential wells [32] on high energy ro-vibration levels [33].

The paper is organized as follows. Section 2 is a reminder of the basic properties of the ozone molecule. Section 3 quickly recalls absorptions in the UV range. Section 4, corresponding to the main purpose of this article, spectroscopy in the infrared range, outlines general trends in theoretical models (empirical and ab initio approaches), experiments and summarizes results of the analyses, separately in two the spectral regions: below 5600 cm^−1^, recorded with Fourier transform spectroscopy (FTS), and from 5600 cm^−1^, up to the dissociation, recorded by cavity ring down spectroscopy (CRDS). Section 5 is devoted to the three above-mentioned topics including sub percent accuracy of line intensities and the questions about the shape of the potential energy surface (PES) in the transition state (TS) energy range. In addition, we show recent unpublished results of the analyses of the ν_3_ band of 18 existing ozone isotopologues with a comparison between observed and predicted parameters (rotational constants and band centers).

## 2. Generalities

### 2.1. Few Words of History

Martinus Van Marum, a Dutch chemist, was probably the first person to detect ozone gas through sensory methods (by the characteristic smell and oxidizing properties that the air acquires after passing electric sparks through it) in 1785. Nevertheless, generally, the discovery of ozone as a chemical (in 1839) is ascribed to Schönbein. He called this gas “ozone”, which is distracted from the Greek word ozein (ὄζειν) meaning “to smell”. Ozone is a highly reactive molecule comprised of three oxygen atoms.

### 2.2. Ozone in the Atmosphere

In natural conditions, the relative concentration of ozone in atmospheric air is very small. The total mass of ozone in the atmosphere is 4 × 10^9^ tons, i.e., 6.4 × 10^−7^ of the mass of the entire atmosphere. The average concentration is 1 mg/m^3^. Despite such a relatively small concentration, ozone plays a key role in protecting life on Earth from ultraviolet solar radiation. The distribution of ozone in the atmosphere is extremely uneven. Its concentration grows with the distance from the surface and reaches a maximum at altitudes of 20–25 km.

The need for systematic studies of atmospheric ozone was quickly realized. During the 1920s, attempts were made to determine the total ozone content in the atmosphere (total ozone column, TOC), first by chemical and then by spectroscopic methods. The determination of TOC was followed by intensive studies of the distribution of ozone in height. Some of the first systematic studies in this area were performed by Gordon M.B. Dobson [34,35,36,37] and Oliver R. Wulf [38,39,40]. Currently, there are entire international communities for measurements related to atmospheric ozone. As noted on the website of the World Meteorological Organization “At present, more than 70 agencies in some 50 WMO Member countries are contributing ozone observations” [2]. Even though these works have been going on for decades, both experimental methods and algorithms for TOC determining are still being improved [41].

#### 2.2.1. Basic Facts on Ozone Formation and Destruction

The mechanism of the formation and destruction of ozone was elucidated in the 1930′s by Sydney Chapman. At wavelengths less than 240 nm, UV radiation can dissociate molecular oxygen O_2_as follows:

O_2_ + *hν* (E > 5 eV) → O + O.

Only a small fraction of solar photons (*hν*) have such high energy, so the amount of the atomic oxygen and ozone produced in the upper atmosphere is relatively small. However, as we reach deeper layers, the number of O_2_ molecules increases and the production rate reaches its maximum between 20 and 30 km. At these altitudes, ozone is produced by collisions between one oxygen atom and one O_2_ molecule, in the presence of another particle (often N_2_ or O_2_), named M:

O_2_ + O + M → O_3_ + M.

Many works have been devoted to the study of ozone formation at the molecular level in fine detail (for example [42,43,44,45,46,47] and references therein), however, many aspects of this complicated problem [48] are not yet fully understood.

Since ozone destruction also occurs in the stratosphere, the ozone concentration depends on the competitive process of formation and destruction. The achieved equilibrium depends on the solar flux, the latitude, and the temperature. This extremely simplified scheme should, in fact, depend on thousands of reactions between most of the molecules present in the atmosphere. In fact, there are several hundred molecules and atoms, leading to thousands of chemical and photolytic reactions, depending on pressure, temperature, and meteorological conditions which lead to vertical distributions that are almost stable within long periods. As for ozone, its main and most important function for life on Earth is to protect plants, animals, and humans from harmful UV rays.

A new interest in the need to study atmospheric ozone arose in the 1970s when the forecasts of Rowland and Molina [49] were found to be justified: the anthropogenic release of chlorofluorocarbons was responsible for the decrease of ozone in the stratosphere. CFC are in principle harmless to ozone (but not to the greenhouse effect), but at high altitude they are destroyed by UV radiation, forming chlorine atoms that react and destroy ozone molecules. This confirmed the hypothesis, which was awarded the well-deserved Nobel Prize in 1974. In fact, they benefited from an unexpected event: the very low stratospheric temperature changed the gas phase into reactions in the solid and gas phase. The new clouds, named polar stratospheric clouds—PSC, of the two forms PSC I and II ([50] and references herein)—dramatically changed the process. Catalytic reactions lead to the destruction of millions of ozone molecules with only one atom of chlorine. These observations of the extremely large ozone depletion are evident proof of the influence of human activity. The need to follow up observations of this destruction has become an absolute necessity.

These observations in the stratosphere are also performed by ground-based measurements, such as the Dobson spectrophotometer [10], and more recently by satellites [3,4,5,6,7,8,9], which cover almost the entire Earth and monitor ozone evolution.

#### 2.2.2. Localization

Ozone is present in the entire atmosphere, from the ground up to about 50 km, with a large variability. Mainly, ozone is concentrated in the stratosphere between altitudes of 10 and 40 km. Although the terms ‘ozone layer’ and ‘ozone hole’ are not strictly appropriate, they had great impact in the media. A straightforward visualization of this concept does not provide a correct image of a real situation, since under normal conditions of temperature and pressure (*T* = 300 K, *p* = 1 atmosphere), the column thickness would be about 3 mm, if it were all squeezed into one layer. This corresponds by definition to 300 Dobson units. The ozone concentration is variable and depends mainly on the latitude and the seasons.

#### 2.2.3. Ozone in the Troposphere

The quantity of ozone in the troposphere represents about 5% of the total amount, but its concentration is very variable depending on the season, day or night time, and the geographic localization in a country side or near big agglomerations. Its increase in the troposphere will never compensate the decrease in the stratosphere, in terms of the absorption of UV radiation. The role of ozone in the troposphere is exactly the opposite of its role in the stratosphere: it is a very strong oxidizer that can be harmful for the health, particularly for the breath. The various chemical reactions in most cases involve nitrous oxides (named NO_x_ and NO_y_) and are associated with air pollution, that is, with anthropogenic impact. A survey of this pollution is widely used, mainly in big cities, and often leads, in particular, to the restriction of car traffic.

In the troposphere, near ground level, ozone molecules are both air pollutants that threaten the health of living things and greenhouse gases that trap heat and contribute to climate change. A small amount of ozone does occur naturally at ground level. Plants and soil release some of it. Some amount of ozone migrates down from the stratosphere. Most of the ozone near the ground is formed because of vehicle exhaust and emissions from factories, power plants, and refineries. Since 1900, the amount of ozone near Earth’s surface has more than doubled due to an increase in industry and the number of automobiles.

Unlike most other air pollutants, ozone is not emitted directly into the air. Tropospheric ozone is formed because of the interaction of sunlight, particularly UV radiation, with hydrocarbons and nitrogen oxides which are emitted by automobile exhaust pipes and smokestacks. In urban areas, high levels of ozone concentrations are usually observed during the warm summer months. Typically, ozone concentrations reach their peak in the middle of the day or in the late afternoon, after exhaust fumes from the morning rush hour have time to react in sunlight. A hot, sunny, quiet day is an ideal environment for the production of ozone pollution. At the end of the day, as the sun starts to set, the ozone production begins to subside. In order to form, ozone needs sunshine to fuel the chemical reaction. When ozone pollution reaches high levels, pollution alerts are issued urging people with respiratory problems to take extra precautions or to remain indoors. When it is inhaled, ozone can damage lung tissues. It is harmful to all types of cells. This can worsen an athlete’s performance or create more frequent attacks for people with asthma, and cause eye irritation, chest pain, coughing, nausea, headaches, and chest congestion. This can worsen heart disease, bronchitis, and emphysema.

Ozone also damages materials like rubber, textile dyes, fibers, and some paints. These materials can be weakened or degraded by exposure to ozone. Some elastic materials can become brittle and crack, while paints and fabric dyes may fade more quickly. Fortunately, some solutions do exist.

What can we do to decrease ozone production in the troposphere? Choosing public transportation, walking, or cycling instead of traveling by car is a good step. If you wait until the evening to refuel your car or mow your lawn, it is unlikely that the pollutants released will become ozone. Also, on a larger scale, you can look for energy sources that do not emit the pollution leading to the formation of ozone. Contact your utility company to find out where your energy comes from.

#### 2.2.4. Ozone Depletion: Observation, Explanations and Solution

As said in Section 2.2.1., various chemical reactions in the stratosphere led to a “natural equilibrium” between formation and destruction for very long periods. However, in 1974, the American chemists Mario Molina and F. Sherwood Rowland [49] of the University of California at Irvine recognized that human-made CFCs molecules containing only carbon, fluorine, and chlorine atoms can be the main source of chlorine in the stratosphere. They also noted that chlorine can destroy a significant amount of ozone after it has been liberated from CFCs by UV radiation. In 1978, TOMS [3] confirmed their hypothesis and in 1985, an extreme depletion of ozone was observed over Antarctica [2].

#### 2.2.5. Ozone and Supersonic Fleet

In the 1990s, a question arose about the possible impact of the supersonic fleet on the destruction of stratospheric ozone. This fleet, mainly represented by the French and English Concorde aircraft, posed a possible danger to the ozone in the stratosphere, since their flight altitude was close to the height of the maximum ozone concentration, and the exhaust of the combustion of kerosene was precisely the creation of NO_x_. A scenario appeared in the scientific literature, in particular that of Johnston [51], which announced an estimated decrease in ozone concentration to 15%. It was the time for Aerospatiale (now Airbus) to make a critical choice for the future (after 1990), which is now the present, between a new supersonic aircraft, named ALLIANCE, and a big aircraft, named A380. Now everyone knows what choice has been made.

## 3. A Few Words on the Ozone Absorption in the UV Spectral Range—General Information

This paragraph is a brief reminder of the ozone absorption of ultraviolet radiation. The reader can find details in the review of J. Orphal ([52] and references therein).

**Figure 1:**
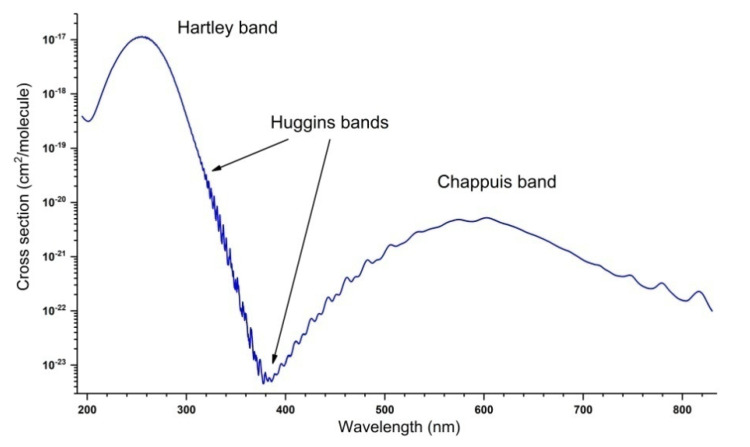
Overview of UV ozone absorption in the spectral range 195–830 nm plotted using absorption cross-section data available in the S&MPO information system (see references in the text).

**Figure 2:**
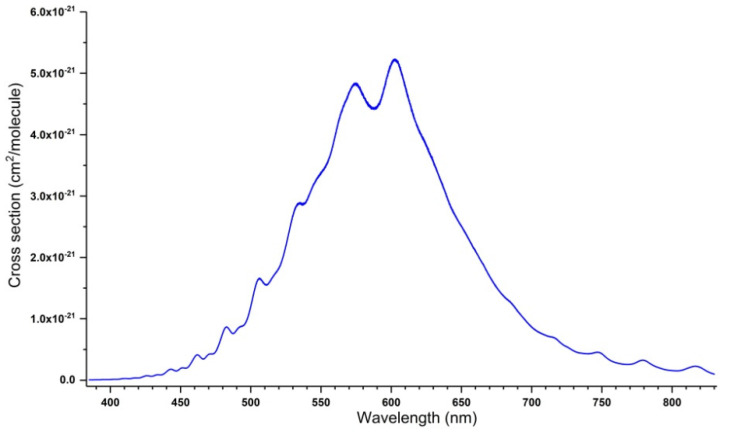
Overview of the Chappuis bands between 385 and 830 nm.

**Figure 3:**
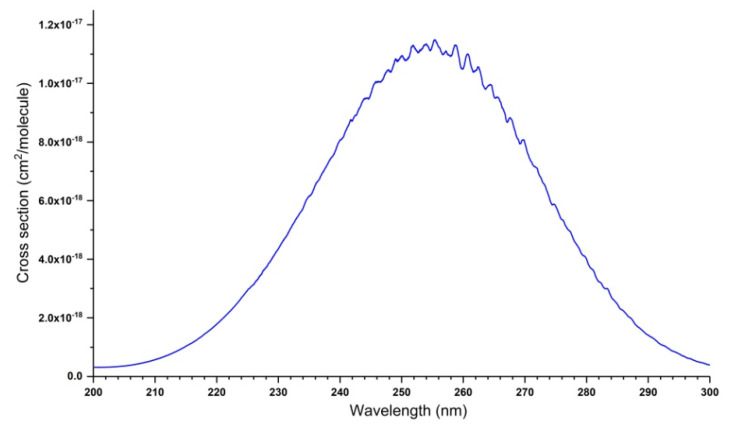
Overview of the Hartley bands between 200 and 300 nm.

The ozone molecule absorbs in most of the spectral regions, from microwave to UV. This feature makes it possible to observe and quantify it, particularly in the infrared range (mainly 10 and 5 microns) and in the UV, in the Hartley band (centered at 255 nm). Other wavelengths corresponding to the Huggins bands near 340 nm or Chappuis bands in the visible region around 610 nm, are also used, but to a lesser extent. Figure 1, Figure 2 and Figure 3 show an overview shape of the absorption cross-sections in the UV and visible ranges. The cross section in Figure 1 is plotted in a logarithmic scale. A large number of publications, documents, and reports recall the main features of these absorption properties [53,54,55,56,57,58,59,60,61,62,63]. The Hartley band, corresponding to the largest absorption coefficient, is often used, particularly in Dobson spectrometers, and now on various satellites with TOMS [3] as a precursor. The room-temperature absorption cross-sections of O_3_ in the Hartley band at 253.65 nm (in 10^−20^ cm^2^/molecule units), measured in various studies, are given in Table 1.

The most recent work [61] recommends the use of the UV reference cross-section value 1123.9 × 10^−20^ cm^2^/molecule at the wavelength 253.65 nm. This new value is used to validate the absorption coefficient in the infrared region (see next Section). The goal of the atmospheric community is to achieve a consistency between absorption intensity values better than 1% for various spectral ranges, in particular for the 10 microns and Hartley bands (most often used for retrievals), but they must also be compatible in both ranges. That was clearly not the case until recently [22,23,62,63].

Many studies in all spectral wavelength ranges over two or three decades were devoted to improving the accuracy in laboratory spectra for extremely accurate quantification in the atmosphere. In this latter case, additional parameters such as temperature or vertical distribution add uncertainties.

The main interest of this short reminder about UV absorption with respect to the next Section is related to the use of the new reference value for the UV absorption coefficient of Hodges [61] to determine the ozone vapor pressure in the cells.

Figure 1, Figure 2 and Figure 3 show the absorption cross-section between 195 and 830 nm at T = 295 K. The data correspond to the spectra recorded by J. Malicet, J. Brion, and D. Daumont in the Groupe de Spectrométrie Moléculaire et Atmosphérique (GSMA) at the University of Reims in the 1970s, using a Jobin-Yvon monochromator, working at a resolution of 0.01 nm, and are provided here as private communication. In addition to the spectrum shown in Figure 1, Figure 2 and Figure 3, these researchers recorded UV spectra at temperatures of 218, 228, 243, and 273 K. The reader can find these spectra in the S&MPO database [64] at https://smpo.iao.ru/xsections (since 1 May 2005). More detailed information is given in [60,65,66,67,68,69,70,71].

## 4. Spectroscopy in the Infrared

This section is the main purpose of this review.

### 4.1. Fundamental Modes and Isotopic Abundance

Quite quickly after the discovery of ozone as a chemical compound, it was found that the molecule consists of three oxygen atoms. However, for a long time it was not easy to determine the geometry of the molecule. Even at the time of the first edition of Hertzberg’s fundamental textbook (G. Herzberg, Infrared and Raman spectra of polyatomic molecules, 1945) the question of the geometry of the molecule and its fundamental frequencies had not been solved unambiguously. Subsequent ab initio studies [72,73,74,75,76] have shown that the electronic ground state potential energy surface has at least four minima. The three lowest identical ones of the C_2v_ symmetry (open geometric configurations) occur due to the Jahn-Teller effect (as discussed by Tannor et al. [77], Garcia-Fernandez et al. [78], and Alijah et al. [79]), whereas the cyclic D_3h_ configuration [80] lies much higher in energy above the first dissociation threshold, as calculated by Siebert et al. [80] and Schinke et al. [81]. The cyclic minimum is separated from the open geometric configurations by large barriers and does not have a noticeable impact on the spectroscopy and dynamics of the molecule in the gas phase. The total symmetry group of the main isotopologue ^16^O_3_ accounting for permutation-inversion operations [82] is D_3h_ (M) as discussed by Lapierre et al. [83].

Since in nature only three oxygen isotopic species with atomic masses of 16, 17, and 18 are stable, then, in principle, 18 combinations of different isotopologues of the ozone molecule are possible (^16^O^16^O^16^O, ^17^O^17^O^17^O, ^18^O^18^O^18^O, ^16^O^16^O^18^O, ^16^O^17^O^16^O, etc.). Mass, natural abundance, partition function, and statistical weigh of 18 possible ozone isotopologues are given in Table 2. However, the C_2v_ (symmetric arrangement of edge atoms) and C_s_ (asymmetric arrangement of edge atoms) species of the same isotopologue could only be separated for the energies deep in the potential wells. Near the dissociation threshold the vibrational states can be delocalized, so that such species cannot be distinguished as shown by Kokoouline et al. [32]. Further, unless otherwise stated, we refer to the ^16^O^16^O^16^O species as the main ozone isotopologue.

**Figure 4:**
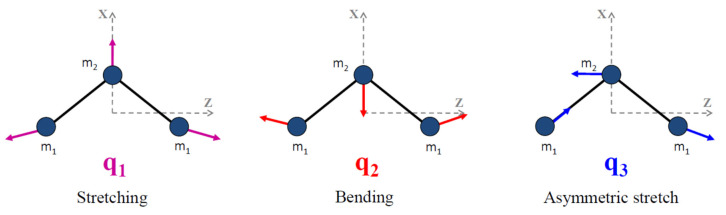
Normal vibration modes of the ozone molecule.

**Figure 5:**
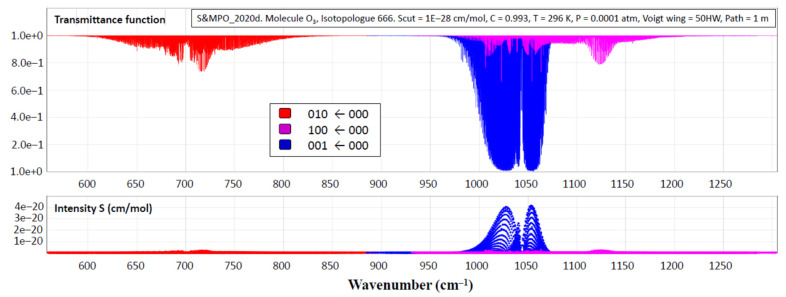
Transmittance functions for the ozone fundamental bands simulated in S&MPO.

In the open geometrical configuration of the electronic ground state, the ozone molecule is an asymmetric top with three vibrational modes. The corresponding fundamental band origins are 701, 1042, and 1103 cm^−1^. The wavenumbers 701 cm^−1^and 1103 cm^−1^correspond to symmetric normal modes *q*_2_ and *q*_1_, respectively, bending and stretching vibrations. The wavenumber 1042 cm^−1^corresponds to the asymmetric stretching mode *q*_3_ (see Figure 4). The vibration states of the molecule are usually labeled by the set of three quantum numbers (V_1_V_2_V_3_), corresponding to the excitation of three normal mode vibrations. The observed spectrum in the infrared region (up to dissociation of about 8500 cm^−1^) corresponds to transitions between various vibration–rotation (VR) states in the electronic ground state. Each vibrational band possesses a rotational structure consisting of hundreds or even thousands of individual VR lines. Transmittance functions for the ozone fundamental bands simulated in S&MPO are shown in Figure 5. These rotational patterns are increasingly complex at a high energy range because of the coupling between various modes. Depending on the intensity cut-off, vibrational bands cover ranges of several tens to hundreds of wave numbers.

One of the most complete resources on the vibrational–rotational absorption spectrum of the ozone molecule is the Internet accessible information-computing system “Spectroscopy and Molecular Properties of Ozone” (S&MPO) at https://smpo.iao.ru and https://smpo.univ-reims.fr (the last release of this database SMPO_20d is accessible since 1 December 2020).

### 4.2. Theoretical Models

#### 4.2.1. Empirical Approach

The analyses of MW and IR spectra have shown that the ozone molecule possess three different rotational constants and belongs thus to the class of asymmetric tops.

Within the traditional spectroscopic approach, which is limited by one potential well in the electronic ground state, the symmetry properties of the ozone ro-vibrational states are usually characterized by the geometrical point group as reviewed by Flaud and Bacis [85]. The “symmetric” isotopic species (having the same edge atoms) belong to the C_2v_ point group, whereas asymmetric ones belong to the C_s_ group. In the spectroscopic literature, it is customary to classify the rotational levels of each vibrational state (V_1_V_2_V_3_) of such molecules by a set of three rotational numbers, *J*, *K_a_*, and *K_c_* [85,86,87,88], where *J* is the quantum number of the total angular momentum, while *K_a_* and *K_c_* correspond to projections on the *a*- and *c*-axes of inertia. Thus, a set of six numbers is used to label the vibration–rotation energy level (the numbers *K_a_* and *K_c_* are often used as subscripts of the number *J*)—(V_1_V_2_V_3_) *J K_a_ K_c_*.

The general form and structure of absorption bands in the spectrum of a molecule are determined by the selection rules for allowed transitions between various vibration–rotation levels. These rules can be formulated either in terms of symmetry species of the full rotational and point groups or in terms of changes in vibration and rotation quantum numbers. For the electric–dipole transitions, one of the main restrictions is the change in the rotation quantum number *J* equal to 0 or ±1. The selection rules for the changing of other quantum numbers in the case of symmetric and asymmetric species are given in Table 3 [85]. Even integer values are indicated by “e” and odd values by “o”.

Since the ^16^O and ^18^O isotopes are bosons with zero nuclear spin, only ro-vibrational levels of A_1_ and A_2_ symmetry types in the C_2v_ point group of the ^16^O_3_, ^16^O^18^O^16^O, ^18^O^16^O^18^O and ^18^O_3_ isotopomers exist:

v_3_ even, *K_a_ K_c_* ee, oo

v_3_ odd, *K_a_ K_c_* eo, oe

The oxygen isotope ^17^O has a nuclear spin of 5/2 that leads to a different situation. The total wavefunction should be antisymmetric (Fermi statistics). The nuclear spin degeneracy for ro-vibrational levels of symmetric isotopic species containing ^17^O atoms is given in Table 4.

##### Calculation of Energy Levels and Transitions

The line lists, which are contained in many spectroscopic databases, usually result from analyses of experimental spectra based on effective models that, in the case of ozone, have been widely described in [85,86,87]. These models are based on the Watson’s type effective Hamiltonian [88] augmented by supplementary coupling terms [89,90], accounting for resonance interactions, and on the effective transition dipole moment operator introduced by Flaud & Camy-Peyret [91].

In most of the studies of high-resolution spectra of the ozone molecule, the rotational (reduced) Hamiltonian for vibrational states is used in the Watson’s form [88].

(1) $$\begin{array}{c} {}^{eff}H^{VV}=E^{VV}+\left[A-\frac{1}{2}\left(B+C\right)\right]J_{z}^{2}+\frac{1}{2}\left(B+C\right)\;J^{2}+\frac{1}{2}\left(B-C\right)J_{xy}^{2}-\Delta_{K}J_{z}^{4}-\Delta_{JK}J_{z}^{2}\;J^{2}-\Delta_{J}{\left(\;J^{2}\right)}^{2}\\ -\delta_{K}\left\{J_{z}^{2},J_{xy}^{2}\right\}-2\delta_{J}J_{xy}^{2}\;J^{2}+H_{K}J_{z}^{6}+H_{KJ}J_{z}^{4}\;J^{2}+H_{JK}J_{z}^{2}{\left(\;J^{2}\right)}^{2}+H_{J}{\left(\;J^{2}\right)}^{3}\\ +h_{K}\left\{J_{z}^{4},J_{xy}^{2}\right\}+h_{KJ}\left\{J_{z}^{2},J_{xy}^{2}\right\}\;J^{2}+2h_{J}J_{xy}^{2}{\left(\;J^{2}\right)}^{2}+L_{K}J_{z}^{8}\;+\ldots \end{array}$$

where $J_{xy}^{2}\equiv J_{x}^{2}-J_{y}^{2}$ and {A,B} = AB + BA. This formula is given in the I^r^ representation and corresponds to the A-type reduction [88].

Due to the approximate equality of harmonic frequencies ω_1_ ≈ ω_3_ and ω_1_ + ω_3_ ≈ 3ω_2_ (see, for example [85]), the entire manifold of vibrational states of the molecule is split into sets of strongly interacting states, for which the basis wavefunctions are mixed by anharmonic and Coriolis interactions. These sets are called vibrational “polyads” [85,87,92] or “resonance groups” [64,93].

The **H^eff^** models of the polyads can be derived by contact transformations [86,94,95,96,97,98,99] for quasi-degenerate states and contain off-diagonal vibrational terms. The first example of such a modeling of strongly interacting states of the ozone molecule was given by Clough & Kneizys [89] when describing the rotational structure of the ν_3_ and ν_1_ bands. To describe the rotational structure of such states, the vibration–rotation effective Hamiltonian (EH) was constructed in the form of an operator-matrix form. In this case the EH for the {(100), (001)} and {(110), (011)} dyads take the form:

(2) $$H^{\mathrm{dyad}}=\left|\begin{array}{cc} H^{V_{1}V_{1}} & h.c.\\ H^{V_{2}V_{1}} & H^{V_{2}V_{2}} \end{array}\right|$$

For the {(002), (101), (030), (200)} tetrad the EH takes the form:

(3) $$H^{\mathrm{tetrad}}=\left|\begin{array}{cccc} H^{V_{1}V_{1}} & & & h.c.\\ H^{V_{2}V_{1}} & H^{V_{2}V_{2}} & & \\ H^{V_{3}V_{1}} & H^{V_{3}V_{2}} & H^{V_{3}V_{3}} & \\ H^{V_{4}V_{1}} & H^{V_{4}V_{2}} & H^{V_{4}V_{3}} & H^{V_{4}V_{4}} \end{array}\right|$$

Here, **H**^V*i*^ (*i* = 1,2,3,4) in equations (2) and (3) are the Watson’s Hamiltonians [88] for single vibration state V*_i_*: (100), (001), (110), (011), (002), (101), (030), or (200). **H**^V*i*V*k*^ (*i*,*k* = 1,2,3,4) are the operators of anharmonic or Coriolis interactions. The notation *h.c.* stands for off-diagonal blocks, which are Hermitian conjugate relative to the lower triangle of the matrix. The general form of the interaction operators **H**^V*i*V*k*^ (**H**^Anh^ and **H**^Cor^) was suggested by Perevalov and Tyuterev in [100]:

(4) $$H^{\mathrm{Anh}}=\sum_{\mathrm{rlm}}F_{\mathrm{LmR}}J^{2l}\{J_{+}^{2r}{(J_{z}+r)}^{m}{+(-1)}^{m}{(J_{z}+r)}^{m}J_{-}^{2r})\}$$

and

(5) $$H^{\mathrm{Cor}}\;\;=\;\;\sum_{r}\sum_{l+m=0}^{r-1}C_{\mathrm{Lmr}}J^{2l}\left\{J_{+}^{r}{\left(J_{z}+r/2\right)}^{m}-{\left(-1\right)}^{m}{\left(J_{z}+r/2\right)}^{m}J_{-}^{r}\right\}\;$$

The indexes V*i* and V*k* in Equations (4) and (5) are omitted. The operators $J_{\mp }=J_{x}\pm iJ_{y}$ are ladder components of the total angular momentum in the molecular frame. This representation has also been used for other triatomic species like water [101] and for rotational sub-groups of polyatomic molecules [102].

The use of EH models for the energy levels is justified by the contact transformations of the ro-vibrational basis set, which must be applied also to the dipole moment operator [86,98,103,104] for the sake of consistency. An effective model for line intensities for semirigid asymmetric top molecules was developed by Flaud and Camy-Peyret in [85,86,87,91]. In practice, the problem is to calculate the matrix elements of the effective dipole transition moment operator between the eigenfunctions of EH (2) or (3):

(6) $${}^{V^{\prime }V}{\tilde{\mu }}_{Z}^{\left(t\right)}\;=\;\;\sum_{k}d_{k}^{\left(t\right)}A_{k}^{\left(t\right)}$$

where V denotes the vibration state (V_1_V_2_V_3_), and $d_{k}^{\left(t\right)}$ are the numerical coefficients determined either from the perturbation theory or by a fitting of computed line intensities to experimental ones. Rotation operators $A_{k}^{\left(t\right)}$ depend on the type (*t*) of vibration band. The first eight operators of expansion (6) can be found in [91]. Later Toth [105] published next eleven terms of expansion (6).

The progress in the recording of absorption spectra imposes increasingly high requirements on the quality of calculations of the VR structure for adequate modeling of these spectra.

##### General Process of Rotational Assignments

**Figure 6:**
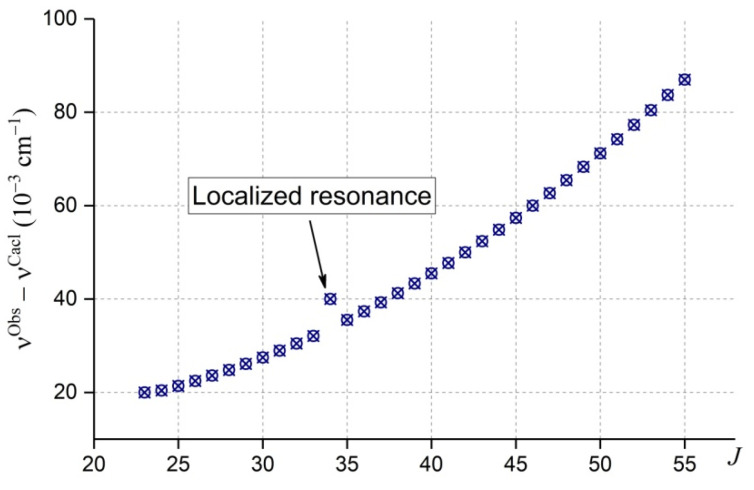
Representation of differences between observed and preliminary calculated positions for a given *K_a_* value.

The spectrum assignment procedure is based on the ground state combination differences (GSCD) explained in a simplified way in the Figure 6. As a rule, the rotational structure of the ground vibrational state (000) is well known, thanks to microwave measurements, the experimental accuracy of which is three orders of magnitude higher than those of typical infrared measurements. As a result, line positions of the transitions reaching the same upper energy level in different branches have precisely the same shift with respect to any (even incorrect) calculation, as shown in Figure 6. Then, the curve (ν^Obs^ − ν^Calc^), as a function of *J* for a certain *K_a_* value, will be similar to the plot of Figure 6. Note that the differences (ν^Obs^ − ν^Calc^) of Figure 6 are given to explain the identification procedure and do not correspond to the real calculation of a certain band. The “ASSIGN” code [106], based on GSCD, has been written to find this type of curve in real spectra and specific calculations. 

**Figure 7:**
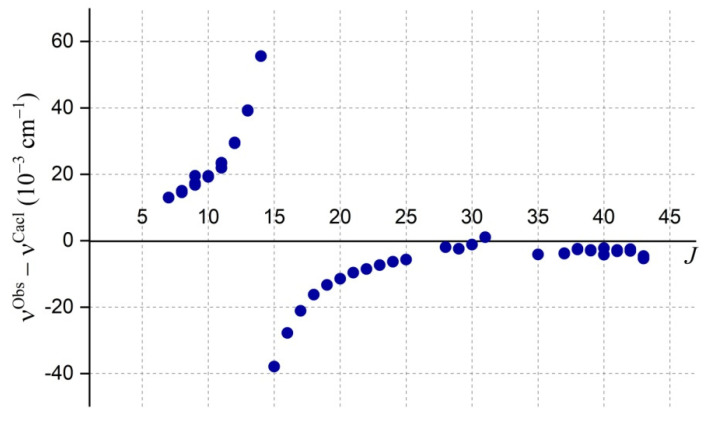
The differences (ν^Obs^ − ν^Calc^) for the ν_1_ + ν_3_ line positions of ^17^O^18^O^18^O as a function of *J* for the *K_a_* = 7 value due to anharmonic interaction with *K_a_* = 7 lines of the 3ν_2_ dark band (see Section 5.2.).

In contrast to the toy example Figure 6, the following Figure 7 represents a real case of the observed intersection of the rotational levels of the (101) and (030) of the ^17^O^18^O^18^O isotopologue (unpublished), that can occur between a bright and a “dark“ state (the definitions of these two latest terms being explained in [21]), leading to a sharp resonance (∆*K_a_* = 3 in this particular case). Such resonances allow information about the band center and the rotational constants corresponding to the dark state to be obtained, even though much less accurately than for the directly observed bands. Obviously, the quantity and quality of useful information directly depends on the number of resonances and their strengths. Each such case is studied in detail in all spectrum analyses. The tables in Section 4.4**. Results** provide examples of such studies. 

The second method of rotational assignment of experimental spectra is the use of synthetic spectra. These spectra are plotted using calculated lists of frequencies and intensities of VR transitions and are directly compared with the observed spectra. For these purposes, we use the MultiFit code developed in the GSMA laboratory [107]. This method has proven its high efficiency, particularly for the assignments of A-type bands in high wavenumber ranges (see Section 4.4. Results).

##### Calculation of Line Intensities

Line intensity *S_ij_* (in cm^−1^/(molecule × cm^−2^) units) for a vibration–rotation transition *ν*_ij_ at a temperature *T* is defined by the Equation (7):

(7) $$S_{ij}\;\;=\;\;\frac{8\pi^{3}10^{-36}}{3hcQ(T)}\;S_{0}g_{c}\nu_{ij}e^{-c_{2}E_{i}/T}[1-\;e^{-c_{2}v_{ij}/T}]\;R_{ij}$$

Here, *c*_2_ = *hc*/*k_B_* with the Boltzmann constant *k_B_*, and *g_c_* and *E_i_* are the nuclear spin statistical weight and the energy of the lower state, respectively. *Q(T)* stands for the partition function, which can be computed using a direct summation of vibration–rotation energy levels weighted with the Boltzmann exponents. The line strength of a dipole-allowed VR transition is defined as $R_{ij}\;=\;\sum MM\prime \langle i|\mu {|j\rangle }^{2}$, where the sum extends over all magnetic sublevels of both the initial and final states.

For direct calculations of intensities, the GIP program [108] uses the method described in Section 3 “line intensities” of reference [91] and also permits operating least-squares fit to experimental data. It was systematically used for analyses of all O_3_ isotopologues in the whole infrared domain included in S&MPO [64] information system.

#### 4.2.2. Ab Initio Calculations for Ozone

##### Line Positions

Empirical approaches to calculate VR line positions using least square fits of the EH parameters to experimental transitions, lead generally to a good agreement between observations and calculations on the order of several thousandths of the wavenumber, at least for vibrational bands up to three or four vibrational quanta. This corresponds to a relative precision in energy levels of 10^−7^ or 10^−8^ that is not nowadays accessible by first principle ab initio calculations beyond very simple molecules with few electrons. However, simplified extrapolations of rotational constants and band centers from empirical models for energy levels above 4000 cm^−1^ are often inaccurate and unreliable. They could be erroneous because of Darling–Dennison resonances [85] and other anharmonic and Coriolis interactions both within each polyad and among overlapping polyads. Fortunately, ab initio calculations in this energy domain can be much more accurate than empirical extrapolations. These calculations have advanced significantly over the years due to the increasing capacity of computers and improved quantum chemistry methods.

The ozone molecule has a complex multi-reference electronic structure. Many efforts have been devoted to ab initio calculations of the potential energy surfaces [72,73,74,75,76,80,109,110,111,112,113,114,115,116,117,118,119] with applications for dynamics [28,29,30,31,45,47,81,112,120,121].

We briefly outline below the works on the electronic ground state PESs since 2000, which explored the shape of the surface near the dissociation or have been used for predictions of vibration levels and wavefunctions.

The first full-dimensional calculation of the PES of the electronic ground state was constructed in the group of R. Schinke, in Gottingen by Siebert et al. [80] using spline representation of a large grid of 5000 nuclear configurations, with one of the bond distances(*r_1_*)varying from 1.9 to 3.3 au, the other one (*r_2_*) from 1.9 to 10 au, and the angle (α) from 60° to 175°. They produced vibrational levels along with some normal mode assignments up to about 7000 cm^−1^, which represented a breakthrough and the reference calculation in the domain at that time. Despite this progress, a drawback was that the Siebert PES underestimated the dissociation energy by about 900 cm^−1^ with respect to the experimental value *D_0_*^exp^ = 8560 cm^−1^ of Ruscic [122], and the accuracy of VR levels were still significantly inferior when compared to the empirically fitted potentials [92,123].

A particular feature of the ozone PES in the context of the applications for molecular dynamics concerns the existence or non-existence of an activation barrier on the minimum energy path (MEP) towards the first dissociation energy. Earlier ab initio calculations [76,80] predicted such a barrier at the transition state between the molecule and the OO + O fragments. Later, more advanced electronic structure calculations by Hernandes-Lamoneda et al. [114] and Fleurat–Lessard et al. [116] suggested that the MEP shape could have a “reef”-like structure [114,116,117,124] with a submerged barrier below the dissociation limit. This “reef” feature was incorporated into a “hybrid PES” [125] by introducing a 1D semi-empirical correction to the 3D surface of Siebert et al. [76]. 

In 2010 Holka et al. [124] explored various PES cuts using a larger full valence and larger basis, resulting in a better agreement for *D_0_* with the observed value. They also provided 3D ab initio calculations of the theory at the MRCI/5Z5 level and calculated force constants for the PES expansion by fitting ab initio energies up to *E*/*hc* = 5000 cm^−1^ above the C_2v_ equilibrium to an eight order---power series expansion of the PES which matched well with empirically determined PES parameters [126].

In 2011, Dawes et al. [112] have shown, by using 1D and 2D cuts of the PES, that including 13 electronic states in the orbital optimization has a big effect on the shape of the electronic ground state potential mainly in the transition state range.

In 2013, Tyuterev et al. [111] proposed a new analytical representation for the 3D ozone PES accounting for its complicated shape on the way toward the dissociation limit. They constructed two PES versions based on extended ab initio calculations. Both PESs were computed at a high level of the electronic structure theory with the MRCI method using the AV5Z and AV6Z atomic basis followed by CBS (“complete basis set”) extrapolation. This first PES obtained, including a single electronic state in the orbital optimization, was called “R_PES” (“reef_PES”), because it maintained a reef structure as had other published ab initio potentials. Another PES reported in [111] was constructed using a more conceptually consistent ab initio approach. The CBS limit *l* = 5, *l* = 6 → ∞ was employed in the entire range of nuclear geometries from the C_2v_ well to the dissociation threshold accounting for 2D “Dawes corrections” [112] on the stretching manifold due to the effect of excited electronic states. This latter potential was called NR_PES (“no_reef_PES”). An RMS error of predictions for the vibrational band origins using NR_PES [111] was about 1 cm^−1^ for various ozone isotopologues measured up to 93% of the dissociation threshold [93,127].

The dissociation asymptotes of both PESs of Tyuterev et al. [111] and of the 3D PES published later by Dawes et al. [113] in 2013 agree well with the experimental threshold value of Ruscic within the uncertainty of about 0.5 %. A comparison of key features of recent ab initio PESs of ozone with respect to experimental data including fundamental frequencies and dissociation energy can be found in Table 1 by Yuen et al. [31] and some other comparisons in the paper by Kochanov et al. [128].

Vibrational levels calculated with the PES of Dawes et al. [113] have been reported in [129,130,131]. A comparison of calculations from the ab initio NR_PES of Tyuterev et al. [111] discussed in next sections shows much better agreement, with (obs-calc) errors typically smaller by an order of magnitude as discussed in [32,33]. This ab initio NR_PES and its empirically optimized version [132] were used for most of the recent analyses of high-resolution spectra. Calculations of vibration–rotational levels from ab initio PESs accounting for all inter-mode couplings, including Coriolis interactions, were reported in [133,134]. Diagonal Born-Oppenheimer corrections to the PES reported by Tajti et al. [135] permitted further improvement of ab initio vibrational levels, at least for the energy range up to half of the dissociation threshold.

There are various important domains of application of ab initio PESs and of variational predictions at high energy ranges. The first one concerns assignments of ro-vibrational spectra in the regions approaching the dissociation threshold. CRDS spectra with very high sensitivity were recorded in this range by the group of Campargue, Kassi, and Mondelain in the LiPhy laboratory (Laboratoire Interdisciplinaire de Physique, Université Grenoble Alpes, France). These measurements reach a noise equivalent absorption, α_min_, on the order of a few 10^−11^ cm^−1^ that was never achieved in high resolution spectra before. This has permitted the observation of very weak lines with intensities down to 3 × 10^−29^ cm/molecule at 296 K. This is three orders of magnitude lower than the weakest lines recorded with the FTS experimental set up and nine orders of magnitude lower than the strongest lines of the ν_3_ band [64]. The CRDS set up was described in detail in [136] (and references therein) and was used for the analyses of many ozone spectra given in [137,138,139,140,141,142,143,144] for ^16^O_3_, [145,146,147,148,149,150] for ^18^O_3_ and [151,152,153,154] for ^18^O–^16^O mixtures of ozone.

Another application concerns the vibrational dynamics near the dissociation threshold [32,33] and the modeling of the isotopic exchange reactions supported by the ozone PES which proved to be very sensitive to the shape of the PES in the transition state range [28,29,30,31] (see Section 4.4.2. Results).

##### Line Intensities

Ab initio calculations of line intensities, in addition to the PES, require knowledge of the dipole moment surfaces (DMS). Earlier DM surfaces of ozone in the electronic ground state were reported by Xie et al. [155] and Diehr et al. [156] in the polynomial representation, which had a limited accuracy. Extended ab initio DMSs with correct asymptotic behavior and intensity predictions across a large range of the overtone and combination bands were reported in 2017 by Tyuterev et al. [133]. Recent comparisons with precise measurements [17] showed its capability to predict intensities for strong lines in good agreement with accurate experimental data simultaneously in microwave, 5, and 10 microns regions. Comparisons with observations (see Section 5.1.for more detail) have also shown that another DMS [157] published in 2018 (using a polynomial model similar to that of [155,156]) underestimated the intensities of the strongest ν_3_ band by about 4%.

The DMS of [133] is currently considered to be the most accurate. It was used for ab initio scaling of the ro-vibrational intensities of thirty ozone bands, subsequently validated by atmospheric and laboratory measurements [158] and included in the last release of the S&MPO and HITRAN databases as described in [18,24]. This DMS was also used for a combined ab initio/empirical line list computed by University College London with collaborators [159].

It is important to recall the following things. The uncertainty in the best variational calculations of the line positions using ab initio PESs is in the order of a few tenths of wavenumber. This represents a significant improvement but is still far from empirical accuracy. When local resonances occur, it is often the case that ab initio predictions might produce larger uncertainties in ro-vibrational patterns. In addition, variational calculations do not always use the same vibrational basis sets as empirical models. An unambiguous assignment is given by (*J*, *Γ*, *N*), where *N* is the global ranking number, and *Γ* is the symmetry type of the upper vibrational state. For these reasons, ab initio line intensities were found useful for predicting the intensities of strong unperturbed lines or for producing general scaling corrections with respect to empirical extrapolations throughout the entire vibrational band. This is explained in detail in [18]. It should also be said that the accuracies are the best in low wavenumber regions (fundamentals and ν_1_ + ν_3_), where they reach the sub percent level. For higher wavenumbers the uncertainty increases, and theoretical predictions do not yet reach experimental accuracy (at least at the moment) for the bands above 5 vibrational quanta, that is beyond 5000 cm^−1^.However, they have two irreplaceable advantages over empirical calculations.

1. They are able to accurately calculate hot bands, which are very often not observed, since they are hidden by stronger bands in the same spectral ranges. In this case their calculation using harmonic oscillator approximation is either very approximate or completely incorrect when Darling–Dennison resonance is pronounced [160].
2. In the case of ozone, when relatively large samples are used (using White cell as an example), ozone decomposition occurs, and its quantification can lead to errors of several percent. In these conditions, scaling factors from ab initio intensities improve the consistency among different spectral intervals [18,158].

### 4.3. Experiments

#### 4.3.1. Ozone Generation

In the laboratory, the generation of ozone is based on the following process: molecular oxygen O_2_ is broken by electric discharges into O atoms and recombines into ozone. This is the principle of standard ozonisors operating in the flow mode. Unfortunately, the performance of this type of O_3_ generator is only a few percent. A way of increasing this percentage was the use of silica gel, adsorbing ozone, which after desorption allowed percentages of ozone up to 30–40% in oxygen to be obtained. The observed color of liquid ozone is dark blue.

In 1978, Griggs performed a new method [161] to generate quasi-pure ozone. This method of ozone generation is described in more detail in [162]. The principle is based on the fact that at the temperature of liquid nitrogen (77 K), the vapor pressures of oxygen and ozone are very different (130 Torr and 10^−3^ Torr, respectively). The applied electric discharge is usually in the order of 4000–7000 volts. Increasing the frequency of the discharge allows for the reduction of the conversion time. This method is relatively fast (a few minutes for works in 10 or 5 microns), but it takes many hours in the case of very weak observed bands with White cells of large volumes. Note that the generation of such large amounts of ozone is hazardous. It is for this reason that the idea of using ozone instead of oxygen to launch space shuttles has been rejected.

#### 4.3.2. Spectrometers

Over the past more than half a century, the spectrometers have undergone major evolution. The first laboratory records for O_3_ were made in 1948 [163] with very low resolution using a Beckman IR-2 spectrophotometer. In 1968, D.J. McCaa and J. Shaw [164] recorded the infrared spectrum using a Perking Elmer 21 instrument equipped with CaF_2_ and NaCl prisms. They determined the band centers with an accuracy of 2 cm^−1^. In 1956, Kaplan [165] recorded the solar spectrum on the Jungfraujoch with a resolution of about 0.02 cm^−1^ and performed a rough analysis that allowed three rotational constants to be determined and led to the conclusion that there is a large Coriolis interaction between ν_1_ and ν_3_. Later, the use of a grating spectrometer, described by J.U. White in [166], made it possible to record the bands ν_3_ and ν_1_ with an experimental full half-width of lines of 0.06 cm^−1^ for ν_3_ and of 0.08 for ν_1_. These recordings allowed individual lines to be identified, despite a much larger experimental width than “natural” (see below). Clough and Kneizys [89] performed the first ro-vibrational analysis and highlighted a strong Coriolis resonance between ν_3_ and ν_1_.

The appearance of tunable diode lasers made it possible to record some isolated lines of the ν_2_ and 2ν_2_–ν_2_ bands in 1979 [167]. The resolution was 0.002 cm^−1^. Unfortunately, this technique covers only a few wavenumbers, which makes it difficult to perform a complete analysis which requires a large spectral coverage. In addition, the scanning of wavenumbers controlled by the current is not completely linear, which requires the use of Fabry Perot and leads to possible errors in positions and intensities.

Later, a large jump was obtained by using Fourier transform spectrometers. The FTS, using the multiplex advantage, was developed in Franceunder the impulsion of P. Jacquinot and P. Connes. The description of the first high-resolution FTS is given in the paper of Connes and Michel [168]. G. Guelachvili, following P. Connes’ principles, built an interferometer permitting a resolution of 0.010 cm^−1^ to be obtained; all details concerning the setups can be found in [169,170].

The first records of ozone high-resolution spectra appeared in the Kitt Peak National Observatory (KPNO) at Tucson (Arizona, USA) and in the GSMA laboratory in the 1980s. This latter was built according the same model as in [169,170] and its characteristics are given in [171,172]. It is important to remember that the accuracy of the line positions for these GSMA spectra varies from 0.5 × 10^−3^ cm^−1^ to 2 × 10^−3^ cm^−1^, depending on their strengths and on the presence of other lines. The main problem concerns the accuracy of the intensities of weak bands. The relative precision, that is, the ratios between transitions in a same polyad, is several percent. Unfortunately, the determination of the exact amount of ozone is very difficult when using White cell filled at a relatively high pressure (50 Torr).

The lines of high-resolution commercial spectrometers have appeared quite recently. High-, medium-, and low-resolution spectrometers designed to solve a wide range of tasks, both research and routine, are offered by a number of manufacturers (BOMEM, Inc. (Quebec, Canada), Bruker (Karlsruhe, Germany), etc.). Most of them work with continuous scanning, like Bruker. They are able to track path differences up to ten meters, that is, they have a resolution of 10^−4^ cm^−1^. Despite the fact that this value is significantly smaller than the natural width (see below), it limits the influence of the apparatus function (instrumental line shape (ILS)). New spectra were recorded with these Bruker instruments in Deutsch Forschungsanstalt Luft & Raumfahrt (DLR), Oberpfaffenhofen Germany, by Birk et al. [173] and Laboratoire d’Etudes du Rayonnement et de la Matière en Astrophysique et Atmosphère (LERMA), Paris France, by Janssen et al. [19] in the range of 10 microns, which lead to a precision of a sub percent for intensities, in agreement with ab initio calculations [17,18,24] (see Section 5.1. Recent Results).

#### 4.3.3. Data Reduction

All research groups have their own software to derive the following experimental parameters: positions, intensities, self-broadening and/or pressure broadening by other gases (for example, air-broadening), and, ultimately, parameters of the line profile when the speed dependence is included [174]. In all cases, the parameters are determined by minimizing the difference (Observed–Calculated) between the observed and calculated transmittances. The fits are performed sequentially for each line, adjusting the experimental parameters. In addition, 100% transmittance is determined (using a polynomial of degree 1 or 2), in a small interval of analysis (between 0.01 and 0.07 cm^−1^, depending on the experimental conditions), and, if necessary, ILS-related parameters. The fits are consistently performed in the whole domain (up to 200 cm^−1^). This procedure can take up to several hours. The program codes obviously take into account the following things:

In these program codes, the line profile is commonly computed as a convolution of the line shapes associated with two physical phenomena: on the one hand, the Doppler effect, leading to a Gaussian shape and proportional to the observed frequency, and, on the other hand, the collisional broadening, proportional to the pressure. Both parameters depend on the temperature. Since this coefficient of proportionality (self- and air-broadening) is on the order of 0.1 cm^−1^/atmosphere, this also leads the line widths of the order of 0.001 cm^−1^ for several tours of ozone.

Both effects are accounted for by the Voigt profile, which was used for all the measurements obtained in the GSMA laboratory and presented in this review. It should be noted that recent progress regarding the line profile, such as the speed dependence, has shown (for example, in the case of CO_2_ [174]) that it is possible to improve the extracted parameters using available measurements. When determining the intensities, it is possible to achieve sub percent accuracy. Nevertheless, Jacquemart et al. [20] have observed that using the qSDVP [175] instead of the Voigt profile did not lead to significant improvements in the case of ozone.

The next step is a convolution using the apparatus function of the spectrometer. Details are given in [19,170,171]. Let us just remind that the FT apparatus function is itself a convolution of the *sinc* function, due to the limited path difference, and the iris function (ILS) of the spectrometer. In the first approximation, the ILS is the Fourier transform of the boxcar function due to the finite optical difference of the interferometer. This corresponds to a *sinc* function which is wavenumber independent. Then, it is necessary to take into account the finite beam size. Details in the case of GSMA FTS can be found in [171,172]. Taking into account the approximate Doppler and broadening values, a path difference of 5 m will be enough for the recordings of the ozone spectra down to 10 micron spectral range. A longer path difference may not lead to a real improvement in the retrieval of experimental parameters (positions and intensities) but it will decrease the signal/noise ratio when using the same recording time.

Another significant improvement in the technique of recording spectra, after the development of FTS, is associated with the appearance of diode lasers. Wenz and Demtröder [176] registered a region of 1.5 microns, which made it possible to obtain spectra with a resolution of several 10^−3^ cm^−1^ and to perform the analysis of the 5ν_1_ + ν_3_ band.

The last breakthrough in accessing higher wavenumber ranges occurred around 1995 with the arrival of the CRDS technique. An excellent experimental set up for recording ozone spectra has been developed in the laboratory LiPhy of the University of Grenoble (France). The reader can find all the relevant details in [136] and references therein. Other details concerning the specific case of ozone are given in [137,138,139,140,141,142,143,144,145,146,147,148,149,150,151,152,153,154]. Let us just recall a few important points in this review. The CRDS technique allows the absorption coefficients, expressed in cm^−1^, to be directly registered. In addition, the width of the apparatus function is completely insignificant compared to the Doppler Effect (0.008 cm^−1^ near 8000 cm^−1^). The experimental accuracy of positions is better than 0.001 cm^−1^. The sensitivity of the CRDS setup is two orders of magnitude higher than those of FTS. The minimum detectable intensities are on the order of 10^−30^ cm^−1^/molecule [33], which is due to the fact that equivalent optical path (*p* × *l*) corresponds to hundreds of thousands of kilometers on Torr. Such a path length is absolutely impossible to achieve with a White cell. This circumstance is crucial to the possibility of recording and analyzing the absorption spectrum of the ozone molecule in the wavenumber domain above 5600 cm^−1^. An additional great advantage of this technique is the small volume of the cell filled with ozone, which makes it possible to record rare isotopic species. For strong lines of stable molecules like CO_2_ or CH_4_, this experimental setup can provide intensity measurements accurate to 1–4% (see for example [174] and references therein). In the case of very weak lines of ozone near the dissociation threshold, the accuracy was evaluated as 10–15% mainly because of well-known issues with controlling partial pressures of this unstable species, which is in dynamic exchange with the oxygen in the cell, as shown in Figure 1 of [33]. However, this is not considered as a significant drawback considering the complexity of correctly modeling the observed spectrum (at least for the moment) in the specified spectral region. Note that, even with this uncertainty, the CRDS measurements provide valuable information on the dipole transition moments of the bands, which are 10^9^ times smaller than the ν_3_ band in the 10 microns range [64].

### 4.4. Results of the Analyses

#### 4.4.1. Ozone Spectrum Below 5800 cm^−1^

One of the first FTS absorption spectra of ozone was recorded using a spectrometer at the Kitt Peak laboratory in the years 1985–1988. The path difference was 1 m, which corresponds to an apodized resolution of 0.01 cm^−1^. These spectra made it possible to record and analyze the bands of ^16^O_3_, reported in Table 5, included in the “*Atlas of Ozone Spectral Parameters*” by Flaud et al. [87], except for the ν_3_-ν_2_ recorded at DRL by Birk et al. [177] in 1994.

Almost two years later, numerous ozone spectra were recorded one after another using homemade GSMA FTS [171,172] with an apodized resolution of 0.0033 cm^−1^. The biggest improvement was the use of a White cell, specially designed to record the absorption spectra of ozone, allowing for a full path length of 36 m and a pressure of up to 45 Torr. Obviously, this permitted new recordings of spectra of all the bands mentioned above in Table 5 to be obtained, significantly expanding the range towards short wavelengths and the number of observed lines. The main result of these studies was the detection and analysis of a number of very weak bands, up to 5800 cm^−1^. General information about the observed bands is given in Table 6.

Next, we report the results of the analysis of absorption spectra in increasing wavenumbers, and in the same spectral region in chronological order. The references to the results of the spectrum analyses below 5800 cm^−1^ are given in Table 6.

The strongest band in the VR spectrum of the ozone molecule is the ν_3_ band. The bands ν_1_, ν_1_ + ν_3_, ν_2_, and ν_2_ + ν_3_is also among the strongest bands of the molecule. Overall, the total intensity of the five aforementioned bands is more than 90% of the intensity of the entire VR spectrum of the ozone molecule. For the first time, the correct vibration band assignment in the IR absorption spectrum between 15 and 3 µm (fundamentals, ν_2_ + ν_3_, ν_1_ + ν_3_, 2ν_1_ + ν_3_ and 3ν_3_ bands) was given by Wilson & Badger [163].

The first study of the rotational structure of the ν_3_ band (9.6 µm spectral range) was carried out by Kaplan et al. [165] based on the analysis of the solar spectrum. One of the first calculations of the VR frequencies of the ν_1_ and ν_3_ bands taking into account the Coriolis interaction was given by Clough & Kneizys [89]. In subsequent years, the calculation of the VR energy levels of the (001) and (100) states were refined and extended (increasing the rotational numbers *J* and *K_a_*) in the works [178,179,180,181], and the parameters of the transition moments reported by Flaud et al. [181]. A very recent work has increased the assignment of *J* and *K_a_* values up to 81 and 23, respectively, with 3741 transitions [21].

The ν_1_ + ν_2_ and ν_2_ + ν_3_ bands of the second dyad of the interacting states (110) and (011) are located around 5.7 µm. The stronger band of the region is the ν_2_ + ν_3_ band. The ν_1_ + ν_2_ band is almost two and a half times weaker than the ν_2_ + ν_3_ band. For the first time, the vibrational assignment of the ν_2_ + ν_3_ band in the absorption spectrum of the molecule was carried out by Wilson & Badger [163] and later by McCaa & Shaw [164]. The first analysis of the rotational structure of both bands and determination of the spectroscopic parameters of the (011) and (110) states were carried out by Jones & Shaw [182]. A more detailed analysis of these bands was performed by Barbe et al. [183] and by Malathy Devi et al. [184]. Transition moment parameters for the ν_2_ + ν_3_ and ν_1_ + ν_2_ bands were deduced from the analysis of 220 measured line intensities by Malathy Devi et al. [184]. The recent work [21] has increased the assigned transitions from 57 to 68 for *J* and *K_a_* from 16 to 19.

The vibrational assignment of the ν_1_ + ν_3_ band (4.75 µm) is given for the first time by Wilson & Badger [163]. Individual lines of this band in the solar spectrum are identified by McCaa & Shaw [164]. The assignment of VR lines up to *J* = 20 in a laboratory spectrum and the determination of the rotational constants of the (101) state were given by Trajmar & McCaa [210]. Maki [211] determined the spectroscopic parameters of the (101) state from an analysis of the ν_1_ + ν_3_ line positions with rotation numbers *K_a_* < 9. The interactions of the ν_1_ + ν_3_ band with 2ν_1_ and 2ν_3_ have not been taken into account in the analysis of [211]. Flaud et al. [185] determined about 1150 VR energy levels of the three vibrational states {(002), (101), (200)} with maximum rotational numbers *J* = 55 and *K_a_* = 16. This allowed the authors of [185] to obtain the spectroscopic constants and parameters of the interaction of the three states. In addition, the parameters of the transition moments of all three bands were determined. The interaction of the (101) state with the (030) state, corresponding to the “dark” band 3ν_2_, was detected by Rinsland et al. [186]. The maximum perturbations in the line positions, exceeding 0.01 cm^−1^, were observed for the lines of the (101) 19_217_ and (101) 45_342_ VR levels. The most extended analysis of the ν_1_ + ν_3_, 2ν_1_ and 2ν_3_ bands was published by Barbe et al. [21].

The next fairly strong bands are ν_1_ + ν_2_ + ν_3_ (centered at 2785 cm^−1^) and 3ν_3_ (around 3046 cm^−1^). The first analysis of the ν_1_ + ν_2_ + ν_3_ band and belonging to the same polyad 2ν_1_ + ν_2_ and ν_2_ + 2ν_3_ bands at low and medium resolution was done by McCaa & Shaw [163] and by Snider & Shaw [212]. Then the 3.6-µm range was investigated at the high resolution of 0.03 cm^−1^ by Meunier et al. [213] and by Barbe et al. [214]. A complete analysis of the triad of interacting states {(012), (111), (210)} was carried out in [187,191]. The high-resolution analysis of the 3.3μm range (the 3ν_3_, ν_1_ + 2ν_3_, 2ν_1_ + ν_3_, and 3ν_1_ bands) was reported by Camy-Peyret et al. [188] and by Bouazza et al. [193]. The most complete joint analysis of eight VR bands between 2600 and 3300 cm^−1^ was performed by Mikhailenko et al. [215]. In this study, in addition to intra-polyad interactions, the Coriolis interactions of the (201) and (111) states with the (130) and (040) states, respectively, were taken into account.

The first rotational analysis of the ν_2_ + 3ν_3_ band was published by Malathy-Devi et al. [216]. Later these results were extended and completed by the analysis of the ν_1_ + ν_2_ + 2ν_3_ band in the same region in [195]. In total, the line positions and intensities, the parameters of effective Hamiltonians, and the moments of dipole transitions for seven cold bands are determined in [194,195,196,217]. The strongest bands between 3350 and 3900 cm^−1^ are the ν_1_ + 2ν_2_ + ν_3_ (around 3450 cm^−1^), ν_2_ + 3ν_3_ (around 3700 cm^−1^), and 2ν_1_ + ν_2_ + ν_3_ (around 3450 cm^−1^) bands. The bands ν_1_ + 2ν_2_ + ν_3_ and 2ν_1_ + ν_2_ + ν_3_were recorded at high resolution and analyzed for the first time in [194,196]. More detailed analysis of the ν_1_ + 2ν_2_ + ν_3_ band in the scheme of four interacting states {(022), (121), (050), (220)} was reported by Barbe et al. [217]. Note that this range also contains the VR lines of 14 hot bands.

The strongest absorption band of the 2.5μm region is the ν_1_ + 3ν_3_ band. Its lines are clearly visible in the atmospheric spectra. The band center of this band was derived from the medium-resolution study by Barbe et al. [218]. Then, using high-resolution spectra, Perrin et al. [219] were able to determine the line positions and intensities of this band. In addition, some line series of the 4ν_3_ and 3ν_1_ + ν_2_ bands were found in the spectra. This allowed the authors to identify the main interactions between the states (103), (004), and (310). Recent reinvestigation of these bands is given in [197].

The integrated intensities of the strongest bands in the range 4050–4530 cm^−1^ are of the order of 1 × 10^−22^ cm/molecule. These bands (2ν_1_ + 2ν_3_, 3ν_1_ + ν_3_, 2ν_2_ + 3ν_3_, and 2ν_1_ + 2ν_2_ + ν_3_) were studied in [198,199,200,201] (see Table 6). The cases of the 2ν_1_ + 2ν_3_ [198] and 2ν_1_ + 2ν_2_ + ν_3_ [201] bands were the simplest as these bands were studied using isolated band models. To describe the 3ν_1_ + ν_3_ band, it was necessary to take into account the Coriolis interaction between its upper vibrational state (301) and the (230) state [199]. The most difficult case was the 2ν_2_ + 3ν_3_ band. For an adequate description of its rotational structure, it was necessary to take into account the interaction of the (023) state with the (122) and (400) states. Note that the two latter states were considered to be dark ones in [200]. In later works [220,221], the lines of the ν_1_ + 2ν_2_ + 2ν_3_ band were identified in the range above 4357 cm^−1^. This information made it possible to determine the rotational constants of the (122) state and significantly improve the quality of the description of both the 2ν_2_ + 3ν_3_ and ν_1_ + 2ν_2_ + 2ν_3_ bands.

As a rule, information about the VR levels of a certain vibrational state (V_1_V_2_V_3_) was obtained from the frequencies corresponding to transitions from the lower ground vibrational state (000) levels. The upper VR levels of all the bands shown in Table 6 were deduced from the analyses of such cold bands. However, in some cases, the cold band may be very weak or even a “dark” one, whereas the hot band corresponding to the same upper vibrational state could be “bright” and significantly stronger. One of these situations, the only one in Table 6, corresponds to the level (302) obtained through the observation of the 3ν_1_ + 2ν_3_ − ν_3_ hot band in the 4070–4140 cm^−1^ region [160].

The ν_1_ + ν_2_ + 3ν_3_ band [202] is the strongest (*S_VR_* = 8.8 × 10^−22^ cm/molecule) absorption ozone band above 4100 cm^−1^. This band is located around 4660 cm^−1^ and strongly perturbed by two quasi-dark ν_2_ + 4ν_3_ and 3ν_1_ + 2ν_2_ bands due to the Coriolis and anharmonic couplings between the (113), (014), and (320) states. Due to the line weakness of the ν_2_ + 4ν_3_ band, most of the information about the rotational energy levels of the upper state (014) was obtained from the analysis of the ν_2_ + 4ν_3_ − ν_3_ hot band in the range 3550–3605 cm^−1^. Examples of numerous resonance interactions between the three states of the group under consideration are given in [202].

**Figure 8:**
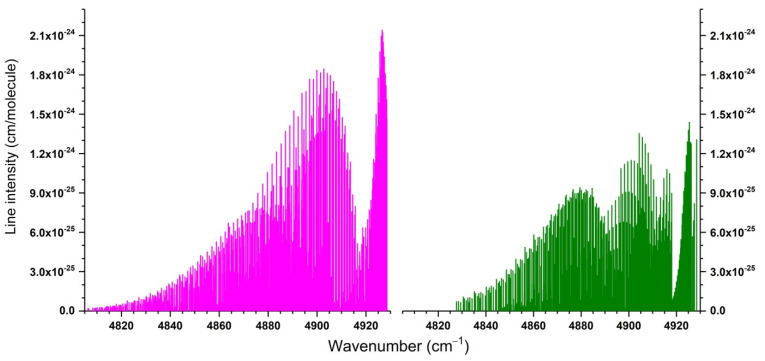
Overview of the calculated 5ν_3_ (left panel) and 3ν_1_ + ν_2_ + ν_3_ (right panel) bands between 4805 and 4930 cm^−1.^

**Figure 9:**
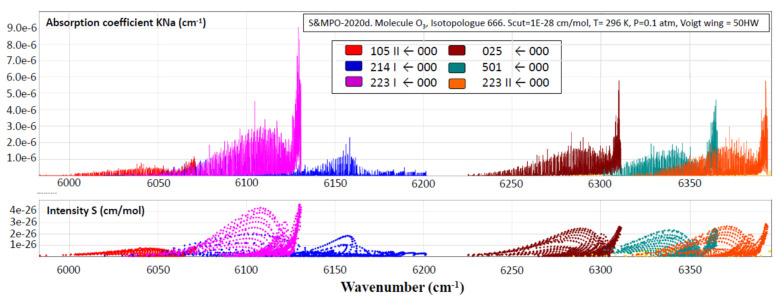
An example of an absorption coefficient simulation of the ^16^O_3_ bands using S&MPO graphical tools based on analyses of the CRDS LiPhy spectra in the range between 6000 and 6400 cm^−1^.

A spectrum analysis between 4850 and 4960 cm^−1^ represented a fascinating puzzle. The observed absorption of the ozone molecule in this region is formed by two bands (5ν_3_ and 3ν_1_ + ν_2_ + ν_3_) of approximately equal intensity and a weaker band ν_1_ + 4ν_3_. Previously, this region was the subject of a band contour analysis [222] from low-resolution spectra. This permitted the band centers for 5ν_3_ and 3ν_1_ + ν_2_ + ν_3_ to be estimated. An advanced analysis of these three bands was published by Flaud et al. [204]. The complexity of the rotational analysis and modeling of these bands is due to the proximity of their vibrational centers: ν_311_ = 4897.3 cm^−1^, ν_005_ = 4919.2 cm^−1^, and ν_104_ = 4922.6 cm^−1^. Strong mixing of wavefunctions leads to the difficulties in assigning a large number of VR levels. For example, the wave function of the *E_VR_* = 5273.261 cm^−1^ level, identified [204] as belonging to (005) 28_326_, has approximately equal contributions from the normal mode basis functions of all three vibrational states; the mixing coefficients are 30% (005), 31% (104), and 39% (311). Strong mixing of VR levels also leads to a redistribution of the intensities of many observed lines to such an extent that even the appearance of the band contour changes. If the contour of the 5ν_3_ band (left panel of Figure 8) is typical for the A-type bands, then for the 3ν_1_ + ν_2_ + ν_3_ band (right panel of Figure 9), this is not the case at all. Similar to the situation with (014) (see the previous paragraph), the hot band ν_1_ + 4ν_3_ − ν_3_ in the range of 3820–3900 cm^−1^ was analyzed to identify the lines belonging to the upper state (104).

The analyses of the 2ν_1_ + ν_2_ + 2ν_3_ (4700–4840 cm^−1^) [203], 2ν_1_ + 3ν_3_ (5000–5100 cm^−1^) [205], ν_1_ + 2ν_2_ + 3ν_3_, 4ν_1_ + ν_3_ (5250–5350 cm^−1^) [206], and 2ν_1_ + ν_2_ + 3ν_3_ (5480–5530 cm^−1^) [207] bands were carried out within the framework of the dyad of interacting states. The integrated intensities of all these bands are less than 1 × 10^−22^ cm/molecule (except for the 2ν_1_ + 3ν_3_ band). All of them relate to cases of high-order resonance interactions which do not follow from the proximity between the harmonic frequencies of the molecule but are due to accidental coincidences of the ro-vibrational energies of highly excited states.

More complex cases are the bands ν_1_ + ν_2_ + 4ν_3_ (5440–5570 cm^−1^) and ν_2_ + 5ν_3_ (5620–5705 cm^−1^) [208], and ν_1_ + 5ν_3_ (5710–5790 cm^−1^) [209]. In the first case, [208], in order to correctly describe the VR levels of corresponding upper vibrational states, it was necessary to construct an effective Hamiltonian for four interacting states {(114), (080), (321), (015)}, taking into account the interactions of the (015) state with all the others. The second case was limited to a triad of interacting states including Coriolis interactions (105) ↔ (006) and (105) ↔ (312). The band ν_1_ + 5ν_3_ (*S_VR_* = 4.9 × 10^−23^ cm/molecule) is at the highest edge of the range that we were able to record and analyze using FTS technique.

The final results of all the analyses described in this section are empirical values of VR energy levels, fitted parameters of effective Hamiltonians and of effective transition moment operators and, finally, complete line lists computed from these parameters. The calculated lists include transition frequencies (ν*_ij_*) and intensities (*S_ij_*), lower energy values (*E_low_*), and the quantum identification of upper and lower levels. Further, these lists, completed by the broadening (γ_air_, γ_self_) and shifting (δ_air_) parameters of spectral lines, are included in spectroscopic databases, such as the S&MPO [18,21,64], GEISA [223], and HITRAN [24,224,225] databases. Note that the calculated lists include a much larger number of bands than is shown in Table 6. First of all, these are weak bands of the corresponding groups of interacting states and hot bands. Using the obtained parameters of effective Hamiltonians, the transition frequencies of hot bands are calculated. In cases where the parameters of transition moment operators of the hot bands cannot be determined from experimental data, these parameters are estimated from those of the corresponding cold bands. For example, the transition moment parameters of the ν_1_ + ν_2_ + ν_3_-v_2_, ν_1_ + ν_2_ + ν_3_ − ν_1_, and ν_1_ + ν_2_ + ν_3_ − ν_3_ hot bands can be estimated using the transition moment parameters of the ν_1_ + ν_3_, ν_2_ + ν_3_ and ν_1_ + ν_2_ cold bands, respectively. Unfortunately, such extrapolations would be valid only in the cases where resonance interactions in all successive polyads corresponding to vibrational excitations are included in a consistent way. However, in some cases, this approach can lead to significant errors in the values of the estimated parameters. One such example is the estimation of the transition parameter of the 3ν_1_ + 2ν_3_-ν_3_ band based on the parameter of the 3ν_1_ + ν_3_ band [160]. The examples of such an inconsistency between cold and hot bands for the ozone molecule are discussed in [160,226].

#### 4.4.2. IR Spectrum from 5800 cm^−1^ towards the Dissociation Threshold

The reason why the results of the spectrum analyses above 5800 cm^−1^ are presented in a separate section is as follows. Above this energy value, vibrational assignment becomes very difficult. The applicability of the simplified polynomial expansion for these purposes becomes debatable, first of all because of multiple anharmonic resonances, including the Darling–Dennison resonance, which is the most familiar one. In addition, the number of states for which the transitions were not observed (dark states), but play a key role due to various resonances, becomes very large. In this energy range, theoretical predictions using the numerical exact variational method based on the potential energy surface become critically important.

As mentioned earlier, it becomes increasingly difficult to analyze the spectra for these energies without reliable predictions of the rotational constants and band origins, and it is almost impossible to carry out such an analysis near the dissociation limit. In addition, such predictions are necessary to account for numerous resonances (anharmonic or Coriolis types) that should be included in the analyses. The second circumstance is the extreme weakness of the absorption lines of this spectral range, typically of the order of 1 × 10^−26^–1 × 10^−27^ cm/molecule and lower. The integrated intensities of the bands do not exceed 1 × 10^−23^ cm/molecule. For this reason, the CRDS technique mentioned in Section 4.2.2. was used to register the corresponding spectra [137,138,139,140,141,142,143,144,145,146,147,148,149,150], unlike FTS used in the lower frequency region.

Below we present a summary of the analyses of the spectra registered with the CRDS setup developed in the LiPhy laboratory of Grenoble University [136]. In total, the main characteristics extracted from the analyses are shown in the following Table 7, Table 8, Table 9, Table 10, Table 11 and Table 12. Alike in Figure 8, for FT region results, we give an example of the calculated spectrum corresponding to the CRDS range.

Table 7 shows a comparison of the experimental (column 2) and calculated (columns 4 and 6) band centers for the ^16^O_3_ molecule. Corresponding calculated values are taken from [132] (Prediction_1) and [111] (Prediction_2), respectively. All numerical values of Table 7 are given in cm^−1^ units. As can be seen from the table, Prediction_1 gives slightly better agreement with experiment because of empirical “fine tuning” of the corresponding PES [132] using observed band origins of ^16^O_3_ below 5000 cm^−1^. The difference between observation and calculation does not exceed 1 cm^−1^ up to 7500 cm^−1^. The root–mean deviation is 0.72 cm^−1^ for the calculation of [132] and 1.17 cm^−1^ for ab initio calculations of [111]. Theoretical values of band origins and rotational constants above 8000 cm^−1^ up to the dissociation energy computed from the ab initio NR_PES [111] are given in [32].

Table 8 shows a comparison of the v-dependent rotational constants *A*, *B*, and *C* obtained from fitting the observed VR line positions of various bands in the range from 5900 to 7860 cm^−1^ with the corresponding calculated values computed from the PES of [132]. The rotational constants *A*, *B*, and *C* in Table 8 are given in cm^−1^ units. As can be seen from that table, in general, the accuracy of the calculation is within 1%. Only for one band (2ν_1_ + 4ν_2_ + 3ν_3_) does the difference between the empirical and calculated values of the *A* constant exceed 1%. Similarly, this difference is greater than 1% for two bands (ν_1_ + 5ν_3_ and ν_1_ + 5ν_2_ + 3ν_3_) in the case of the *B* constant and for one band (5ν_1_ + 2ν_2_ + ν_3_) in the case of the *C* constant. The root-mean deviations are 0.42%, 0.58%, and 0.56% for the *A*, *B*, and *C* constants, respectively.

The highest ν_1_ + 6ν_2_ + 3ν_3_ rotationally resolved band at 7968.78 cm^−1^ observed and assigned in CRDS spectrum [33] is a particular case, because the calculated band origin was found to be sensitive to the effect of delocalization (Kokoouline et al. [32]) of the vibrational upper state wavefunction among three potential wells. Theoretical prediction, which resulted in an Obs-Calc deviation of −2.9 cm^−1^ in the one-well model was slightly improved to −2.3 cm^−1^ when using full-symmetry calculations involving three wells [32] with the same ab initio PES [111].

Predictions for the v-dependent rotational constants were carried out using the PES of [132].

Table 9 and Table 10 are the same as Table 7 and Table 8 but concern the ^18^O_3_ molecule. The comparisons in both Table 9 and Table 10 are given only to the calculation of [132]. As can be seen from Table 9 for ^18^O_3_, the band center calculation reported in [132] has approximately the same accuracy as for ^16^O_3_. The maximum deviation Δν is 2.49 cm^−1^ for the 2ν_1_ + 8ν_2_ + ν_3_ band. The root-mean deviation is 0.85 cm^−1^ with a maximum (ν^Obs^ − ν^Calc^) deviation of 2.69 cm^−1^ for the 2ν_1_ + 8ν_2_ + ν_3_ band at 7908.84 cm^−1^. The same conclusion can be drawn to the quality of calculation of rotational constants (see Table 10). For all bands, the difference between observation and calculation is within 1%. The root-mean deviations are 0.32%, 0.39%, and 0.48% in the *A*, *B*, and *C* constants, respectively.

The results of comparison of the experimental and calculated [132] values of the centers of bands and rotational constants *A*, *B*, and *C* for other ^18^O-enriched isotopic species are given in Table 11 and Table 12; they are organized similarly to Table 9 and Table 10 and for ^18^O_3_.

Two general remarks for the analysis of the spectra of isotopic modifications containing both ^16^O and ^18^O, in addition to ^16^O_3_ and ^18^O_3_: The analysis of these spectra is much more difficult due to the following reasons. First, the method of ozone generation does not allow the different isotopic ozone species to be experimentally separated. Second, the ^16^O^16^O^18^O and ^16^O^18^O^18^O species, which belong to the C_s_ point group, have more symmetry allowed transitions due to non-vanishing spin weights of all VR levels (see Section 4.2.1.). Third, there are a larger number of possible anharmonic couplings between vibrational states, because they all correspond to the same symmetry type A’ of the C_s_ point group. From the theoretical point of view, estimation of the intensity distribution in different branches of the bands is not yet sufficiently accurate in many cases. All this leads to a significant complication of the spectrum and considerable difficulties in the vibrational–rotational identification of individual lines.

Another difficulty is related to the vibrational assignment in terms of normal modes. As mentioned above (see Section 4.2.2.), many predictions from ab initio PESs do not use normal mode basis sets. Variational predictions provide a global assignment (*J*,* Γ*,* N*) where *Γ* is the irreducible representation of the symmetry group and N is the ranking number. Due to the high density of the vibration levels in the energy range above 6000 cm^−1^, an ambiguity in the normal mode vibrational assignments is possible. Non-uniformity in the formation of a group of interacting states, when dark states are included in the fit of effective Hamiltonians to improve the root mean square deviation (*RMS*), can also be a source of additional errors.

**Figure 10:**
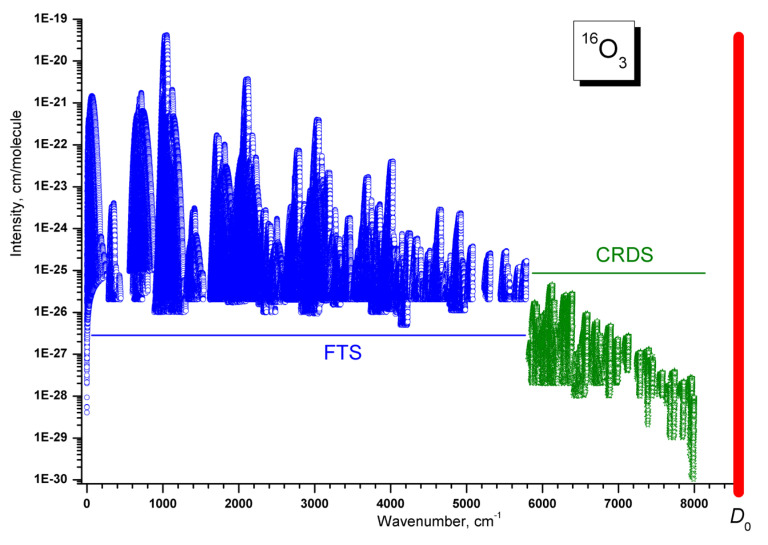
Stick-intensity diagram corresponding to ozone (^16^O_3_) spectra studied to date. The FTS data are available in S&MPO, HITRAN, and GEISA spectroscopic databases complemented by MW data at low wavenumber range. The diagram at the CRDS range corresponds to figures of [33,127].

As a summary of this section, Figure 10 shows a general view of the ozone (^16^O_3_) stick-intensity spectrum studied to date. The spectrum studied to date reaches the region of 8000 cm^−1^, about 93% of the dissociation threshold *D_0_*^exp^ = 8560 cm^−1^ [122,124]. There is a significant decrease in the intensity of the bands above 5800 cm^−1^, which once again demonstrates the advantage of recording these spectra by the LiPhy CRDS setup [33] and of using theoretical predictions for the analyses.

**Figure 11:**
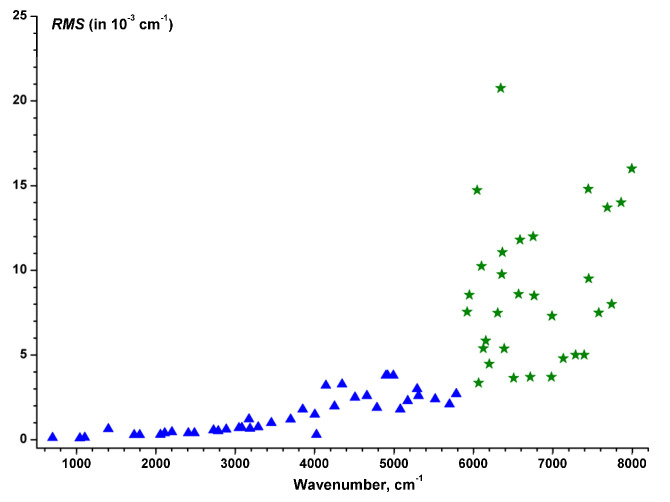
Root-mean-squares (ν^Obs^ − ν^Calc^) deviations of the fits of vibration–rotation line positions using EH models with the increasing wavenumbers of the band origins.

As mentioned above, the results obtained in the studies presented here (ν*_ij_*, *Σ_ij_*, *E_low_*, VR assignment), augmented with additional parameters (γ_air_, γ_self_, δ_air_) and coefficients of their temperature dependence (at least in the range from −70 °C up to +50 °C), are incorporated into spectroscopic databases (for example, S&MPO [64], GEISA [223], and HITRAN [24]). The use of spectroscopic bases for various atmospheric tasks, such as the simulation of atmospheric absorption and transmission spectra, determination of the total molecular column and altitude profiles of molecular concentrations, radiative transfer in the atmosphere, radiation blocks of climate models, etc., places increasingly high demands on the accuracy of the parameters of these databases and their completeness. As a rule, the data presented in the databases are a mixture of observations and calculations. Like in the case of line intensities of the ozone molecule, only a small part of the line broadening coefficients can be determined from the observed spectra. Then, it is necessary to use empirical models to interpolate or extrapolate to all quantum numbers that may be necessary for atmospheric tasks. One example of experimental measurements is given in [180], and the calculation is given in [84]. The HITRAN database [24] provides individual references to the source for each of the six parameters of each transition: position, intensity, air- and self-broadening, air shift, and temperature dependence of the air-broadening. Unfortunately, empirical models have difficulty in producing calculations within experimental accuracy. This problem is discussed in detail in recent papers for both intensities [18] and positions [21]. In the case of line positions, the accuracy of calculations degrades due to an increase in the number of interaction terms, either with dark states or corresponding to accidental resonances. Figure 11 demonstrates the variation of the root-mean-squares (ν^Obs^ − ν^Calc^) deviations for ^16^O_3_ data fittings versus wavenumbers. As can be seen from the picture, the *RMS* deviations increase sharply starting from about 4000 cm^−1^. This is explained by the fact that ″truncated″ models of Hamiltonians (usually including 3 or 4 interacting states) are used for VR levels fitting, taking into account only the main, most significant resonances. The use of simplified models is due to the fact that there is no empirical information about many dark vibrational states and their parameters cannot be estimated with sufficient accuracy. These circumstances lead to deterioration in the quality of the description (an increase of *RMS* deviations).

A particular case of a very highly excited state assigned as (1.6.3) was discussed in [33]; the largest value of the corresponding *RMS* deviation ~0.075 cm^−1^ is due to the issues of the applicability of the conventional EH model in this energy range [32].

**Figure 12:**
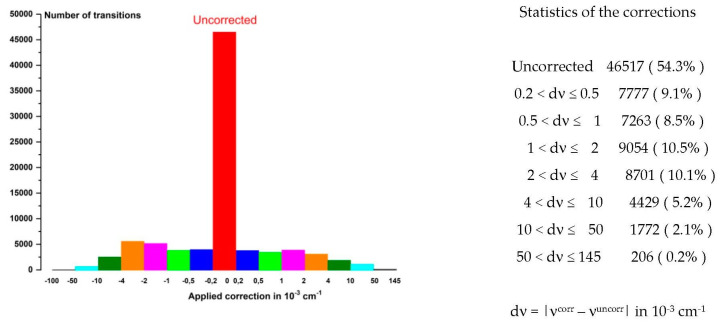
Number of corrected (ν^corr^) and uncorrected (ν^uncorr^) line positions.

One of the ways to obtain more accurate values of line positions in databases is to replace the calculated transition frequencies with observed values (or with the difference in empirical energy levels obtained from the observed line positions) when the differences will be greater than some chosen value (for example 0.001 cm^−1^). In a recent paper [21], this was done for the S&MPO database [64] and will be integrated in the two other databanks [24,223]. Note that these changes correspond to long and tedious work, since the same change must be applied to all transitions of the same upper energy level. This corresponds to three transitions in the *A*-type band and six in the *B*-type band. In addition, this change should also be made for hot bands, in particular, having the (010), (001), (100), and (020) states as lower ones. The statistics of these changes made for the S&MPO database are summarized in Figure 12, as given in [21]. Corrected (ν^corr^) and uncorrected (ν^uncorr^) line positions correspond to the SMPO2020 and SMPO2017 line lists, respectively.

## 5. Recent Results

All the comparisons with experimental observations reported in this section are based on variational predictions using the ab initio PES of [111] or the empirically optimized PES of [132] together with intensity calculations using the ab initio DMS of [133].

### 5.1. The Subpercent Accuracy of the Line Intensities in the Fundamental ν_3_ Band (10 Microns) and the Combination ν_1_ + ν_3_ Band (5 Microns)

As mentioned above, the ν_3_ and ν_1_ + ν_3_ bands are the strongest bands of the absorption spectrum of the ozone molecule in the 10 micron ad 5 micron ranges. Their contributions are 77% and 6.7%, respectively, of the full intensity of the VR spectrum. The maximum intensities of individual lines reach 10^−20^ cm/molecule in the ν_3_ band and 10^−21^ cm/molecule in the ν_1_ + ν_3_ band. For this reason, errors in determining the absolute line intensities of these bands, even by a few percent, can be critical for many applications. The purpose of the studies described in this section was to determine the absolute intensities of the absorption lines of mentioned bands with an accuracy of at least 1% or better.

For the ν_3_ band, the goal has been achieved through a joint study of three experimental groups [19,20,21,22,23] and theory, using the DMS of [133].

The first experiment was obtained using a new experimental setup in the LERMA laboratory [19]; using a Bruker IFS 125 permitted almost simultaneous recordings at 10 μm (*L* = 5 cm) and 5 μm (*L* = 20 cm). The ozone pressure was continuously monitored by UV absorption at 253.65 nm. A multi-spectrum fitting procedure using a speed-dependent line profile model was used to retrieve 740 and 513 line parameters at 10 μm and 5 μm, respectively, based on the analysis of four spectra at ozone pressures between 0.14 and 1.16 Torr. The results have been presented at the HITRAN meeting [227]. The second experiment, performed in the DLR laboratory, has been presented in the HITRAN meeting [62] and published in the ESA report [22]. These results were also obtained using a Bruker instrument. The third experiment, corresponding to a work performed in the GSMA laboratory, was also presented at the HITRAN meeting [23] and is currently published [21].

The weighted fittings of the retrieved line intensities within the framework of the polyad model, and the method of effective operators leads to standard deviations (*RMS*) close to experimental precision. Extensive tests show that the fits are very reliable with respect to the number of adjusted parameters and to the data weighting. The obtained deviations due to the model fit are in the range of about 0.1–0.3%. The comparisons show excellent agreement between ab initio calculations [17,133] and new empirical line lists. Average deviations for strong and medium lines are within 0.5% or 1%, corresponding to uncertainty estimates for both theoretical and experimental results. 

For 5 microns, a comparison was made between the ab initio predictions [17] and the experimental results of the LERMA [20] and GSMA [21] laboratories. The modeling of GSMA experiments with ab initio intensity corrections led to the final line list published in the S&MPO database [18] and partly included in the last release of the HITRAN database [24]. The comparisons confirming the sub percent level of accuracy of the obtained intensities are given in [17,18,24] and were presented at the AGU meeting in Vienne [63]. Table 13 shows the main results of these comparisons.

**Figure 13:**
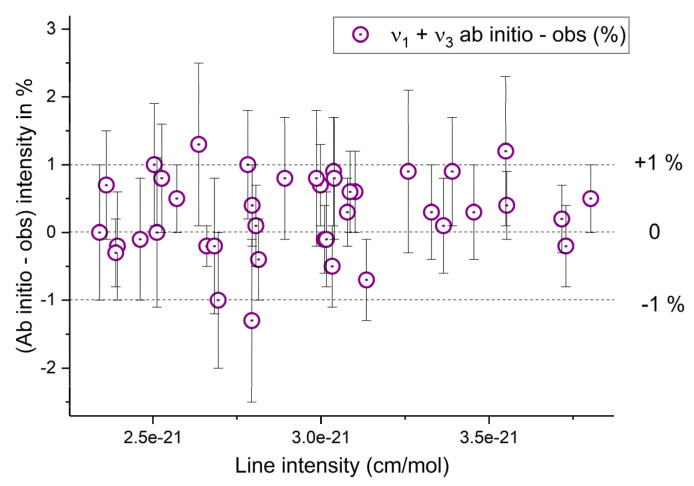
Intensity deviations between ab initio intensities [18] and the sample of accurately measured intensities in the GSMA spectra for the 5 micron range.

Two examples showing the good agreements are presented in next two figures. The first one is Figure 13, which shows the intensity comparisons between ab initio calculations [18] using the DMS of [133] and the sample of very accurately measured intensities in the GSMA spectra for 5 microns. As can be seen from the figure, most of the discrepancies lie within 1%.

**Figure 14:**
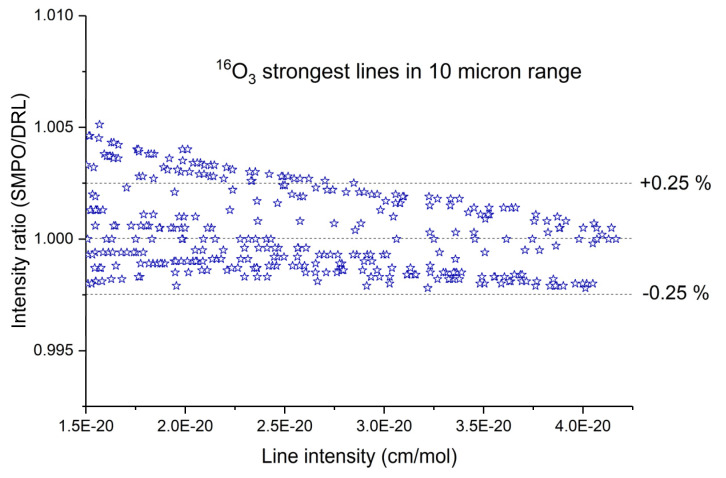
The intensity ratio of the S&MPO [21] and DRL [173] data sets for strong lines in the region of 10 microns as reported in [24].

The second example (Figure 14) shows the comparison of strong-line intensities between the DRL [22,173] and SMPO_20d [21] line lists based on the corresponding experiments in the 10 micron range. As observed in the figure, the differences are less than 0.25% for the strongest lines and do not exceed 0.5% of the measured lines above the cutoff of 1.5 ×10^−20^ cm/ molecule.

### 5.2. Results of the ν_3_ Band Studies of the Eighteen Isotopic Species of the Ozone Molecule

As noted above, the ν_3_ band is the strongest one in the spectrum of the ozone molecule. This fact makes it a convenient and well-implemented tool for the study of rare isotopic modifications. To date, the ν_3_ band has been analyzed for thirteen isotopic species among the possible eighteen. Our goal is to identify the ν_3_ band lines for the five remaining isotopologues: ^16^O^17^O^18^O, ^16^O^18^O^17^O, ^17^O^16^O^18^O, ^17^O^17^O^18^O, and ^17^O^18^O^17^O. All of them but one (^17^O^18^O^17^O) are asymmetric and belong to the C_s_ point group. The first three species consist of three different oxygen isotopes and occupy the 8th, 9th, and 10th places, respectively, following the decreasing natural abundance of the ozone molecules (see Table 2). The proportion of each of them is about 15 × 10^−6^ in nature with respect to the main isotopologue.

**Figure 15:**
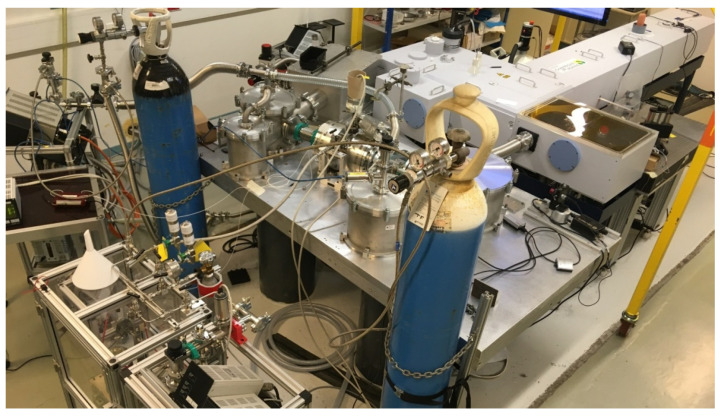
New spectroscopic facilities in in GSMA laboratory of Reims University.

For this purpose, we recorded the spectra in the GSMA laboratory using a new Bruker 120 FTS in the range from 800 to 1300 cm^−1^. Figure 15 shows the experimental setup and the ozone generator described in Section 4.3. Experiment. Two samples with concentrations given in Table 14 were used to register the spectra. This permitted the identification of lines of isotopologues of interest.

We succeeded in assignment of the spectra and the final overall results are reported in Table 15. We note that, not only have we been able to analyze the five missing isotopic species, but we have improved the results of eight others, with a much larger set of assigned *J* and *K_a_* quantum numbers, as stated in the note of this table.

The general results of the analyses are presented in Table 15. The table for each isotopologue shows the maximum values of the rotational numbers *J* and *K_a_* corresponding to the transitions of the lines assigned in the spectra; *NT*, the number of assigned transitions; *RMS* (in cm^−1^), root-mean deviation from fitting this set of line positions within the framework of the effective Hamiltonians method; and a reference if these results have already been published. As can be seen from the table, we managed to complete the task of assigning the lines of five previously non-observed species. In addition, we significantly improved the results for eight other previously investigated isotopologues; a much larger number of lines were assigned and the range of rotational numbers *J* and *K_a_*, for which lines were found in the recorded spectra, was extended.

The comments “TW” (this work) in the last column of Table 15 correspond to the improvements compared to previous works listed in the “Ref” column. These improvements are mainly due to an extension of the assigned transitions in quantum numbers *J* and *K_a_*. The rows given in italic and in bold characters correspond to the most recent unpublished analyses.

In Table 16 and Table 17, we present a comparison of the parameters retrieved from the analyses and calculations using the PES of [132]. As can be seen from these comparisons (see Table 16), theoretical predictions of the ν_3_ band centers and rotational constants of the (001) state based on the PES of [132] demonstrate high accuracy, including rare isotopic species. As can be seen from Table 16, the difference *d*ν = (ν^OBS^−ν^CACL^) between the empirical and calculated ν_3_ band centers does not exceed 0.2 cm^−1^, with nearly systematic offset for all isotopologues. All numerical values of Table 16 are given in cm^−1^ units.

Table 17 presents a comparison of empirical and calculated (based on PES, [132]) rotational constants *A*, *B*, and *C*. Relative differences $\Delta P\;=\;\frac{P^{OBS}-\;P^{PRED}}{P^{PRED}}\;\times \;100\%$, (*P* = *A*, *B*, or *C*) are given in percentages (columns 4, 7, and 10). For the *A* and *B* constants, the differences are within 0.8%. The best agreement (less than 0.1%) is for the ^18^O^18^O^18^O isotopologue. A slightly worse agreement between the experiment and the calculation is observed for the *C* constant. In general, the differences lie within 1.6% with a maximum deviation of 1.82% for the ^18^O^16^O^18^O isotopologue. The *A*, *B*, and *C* values are given in Table 17 in cm^−1^ units.

Note that the slightly worse agreement for the *C* parameter is probably due to the high correlation between its value and Coriolis parameters used to describe the interaction between ν_3_ and ν_1_, whereas predictions correspond to single state analyses.

**Figure 16:**
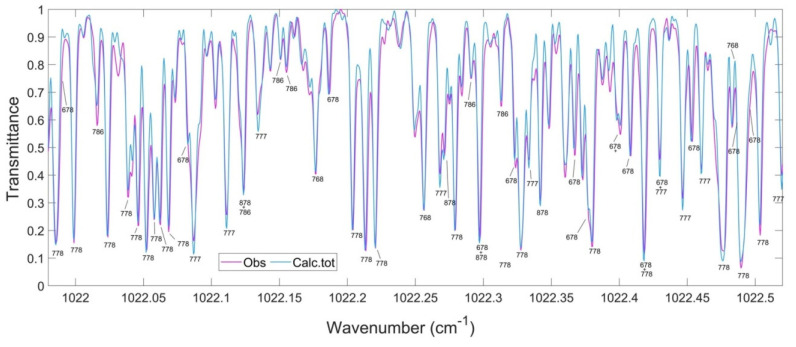
Example of a comparison between recorded (in blue) and simulated (in red) spectra around 1022.3 cm^−1^.

**Figure 17:**
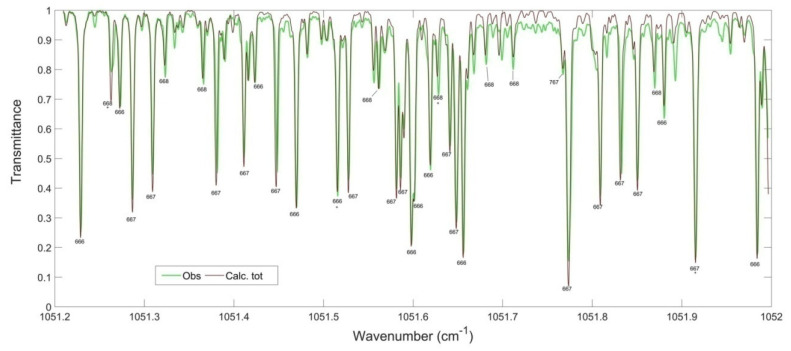
Example of a comparison between recorded (in green) and simulated (in red) spectra around 1051.6 cm^−1^.

The next two Figure 16 and Figure 17, show the agreements between observations and calculations after analyses, fits and creations of synthetic spectra into different spectral ranges. The strong lines of the 1022 cm^−1^ range correspond to the *P* and *Q* transitions whereas those of the 1051 cm^−1^ range correspond to the *R* transitions of the ν_3_ band.

### 5.3. Results of Analyses and Comparison with Theoretical Calculations at Higher Energy Range

#### 5.3.1. Analyses of Spectra as a Tool for Exploring the PES Shape in the Transition State Range

The results of analyses of experimental laboratory spectra at high energies are of major importance to the validation of the potential energy function of the ozone molecule in a wide range of nuclear geometries which supports the collision OO + O processes. The shape of the PES in the transition state range has an impact on isotopic exchange reactions involving the diatomic and atomic oxygen.

**Figure 18:**
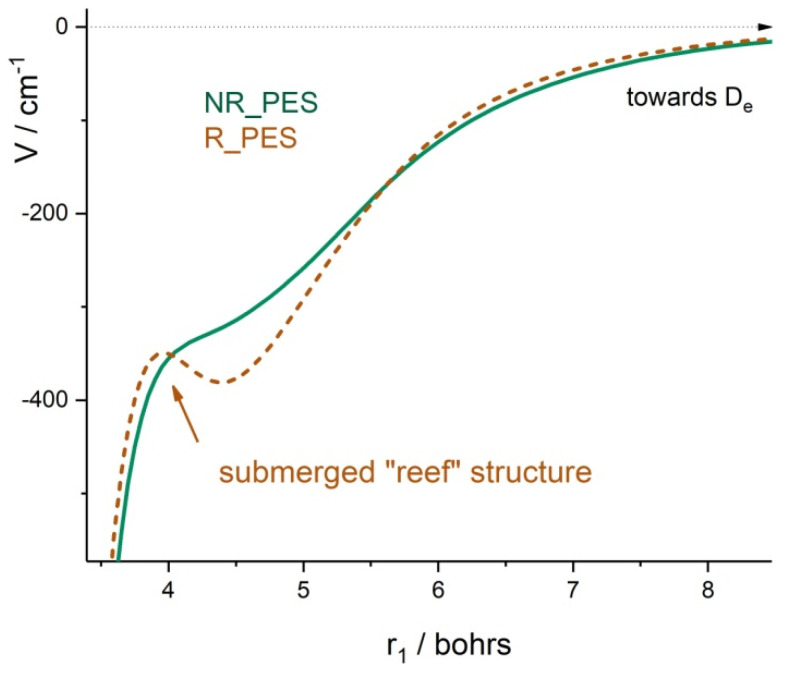
Minimum energy path in the transition state range of two versions for the ozone electronic ground state potential energy function from [111]. The R_PES (“Reef” PES, dashed curve) has the submerged barrier below the dissociation asymptote. The NR_PES (“no-Reef” PES, solid curve) does not possess this feature.

The discovery of the mass-independent fractionation [11] is a well-recognized milestone in the study of the isotopic effect of selective enrichment of heavy ozone isopotomers. This enrichment has been observed in the stratosphere despite the relative accuracy, but was undoubtedly observed in laboratories [11,12,13,14,15]. These phenomena are linked to the isotopic dependence of the ozone formation rates and to the complex energy transfer dynamics near the dissociation threshold. It is well known that there is a strong link between PES properties and dissociation or association. Earlier ab initio electronic structure calculations of the ozone molecule have predicted either an activation barrier [76,80] or reef-like structure [114,116,117,124] (with a submerged barrier below the dissociation, Figure 18) on the minimum energy path (MEP) towards the dissociation. A brief reminder of the issues related to the shape of the PES was given in Section 4.2.2. Schinke et al. [81] have shown that a presence of the barrier leads to a decrease in the rate coefficient of isotope exchange reactions ^16^O + ^18^O_2_→^16^O^18^O + ^18^O by 3–5 times and to their incorrect temperature dependence. On the other hand, Dawes et al. [112] noted that the reef structure could occur as an artifact due to an avoided crossing with excited electronic states. The impact of the barrier or of the reef structure on isotopic exchange reactions has been discussed in many studies [28,29,30,31] (and references therein), where it was found that the smooth PES shapes without these features resulted in better agreement with the experiment for the temperature dependence of the rate constants.

**Figure 19:**
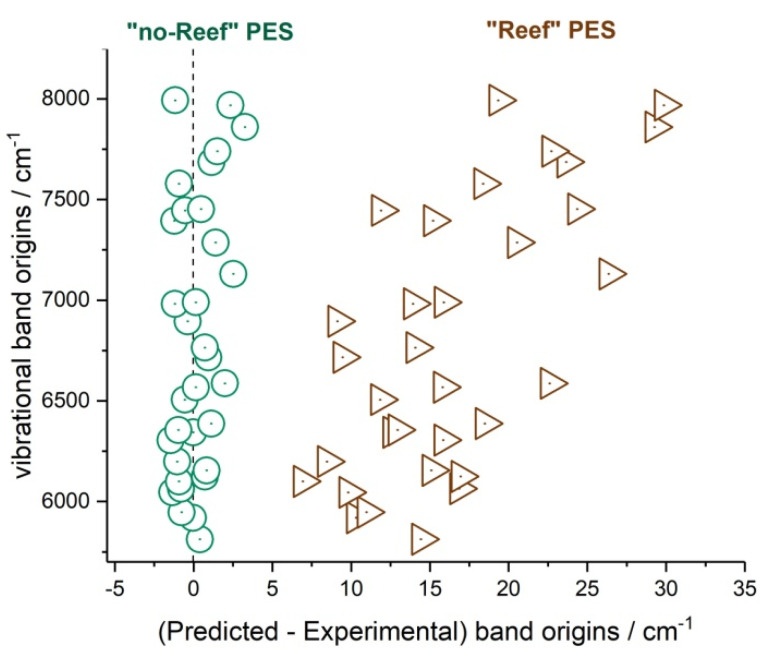
Comparison of the prediction errors using two versions of the ab initio PESs of [111] with respect to experimental band origins in assigned CRDS spectra at high energy ranges. Round green circles correspond to the NR_PES (“no-Reef” PES) and orange triangle to the R_PES (“Reef” PES) which exhibits the submerged barrier below the dissociation asymptote; see the 1D cuts in Figure 18. The diagram is taken from [127] with the two last observed band centers added from [33].

Tyuterev et al. [111] have reported two versions of ab initio PES built up using high-level electronic structure calculations. In the first version (R_PES), the reef structure was present at the transition state range as in earlier calculations [117,124], and the second improved ab initio version (NR_PES) of [111], accounting for Dawes’ finding [112], exhibited a smooth shoulder without barrier at the minimum energy path. The comparison of theoretically predicted vibrational band origins, using the ab initio PESs of [111] with experimental results is reported in [127]. They have found that it was not possible to make a physically meaningful assignment of the CRDS spectra above 6000 cm^−1^ using the calculated levels from the PES which exhibits the reef structure, as illustrated in Figure 18. In contrast, the NR_PES without reef barrier provides excellent accuracy. The *RMS* deviation for all the band centers is less than 1.5 cm^−1^ for PES without a reef, compared to the discrepancies of the order of 10–30 cm^−1^ for the R_PES with the reef structure (see Figure 19). These results corroborate with numerically accurate modeling of the isotopic exchange reactions (reported by Guillon et al. [30], Honvault et al. [31], and Yuen et al. [32]), which showed that the NR_PES of [111] currently provides the best agreement with these chemical experiments as well.

A recent work by Vasilchenko et al. [33], reporting the analysis of the 6ν_1_ + ν_2_ + ν_3_ of ^16^O_3_ with a good agreement between observations and predictions (see Figure 19) confirmed this conclusion (the advantage of PES without reef) by extending the observations to the energy of 8277 cm^−1^ for the highest vibration–rotation level (611) 25_322_ assigned via the observed transition, which is about 96.7% of the dissociation threshold.

Finally, in the most recent analysis of the 7ν_1_ + ν_3_ band of ^18^O_3_, the highest so far assigned vibration–rotation level (701) 27_720_ was reached by an observed transition in the CRDS spectra [150]. The energy of this “highest” level is 8355.3 cm^−1^, corresponding to 96.9% of the dissociation energy for ^18^O_3_, which is theoretically estimated at about 8620 cm^−1^. A study of heavy isotopologues permits accessing previously unknown combination states as shown in the recent work [150]. For the main isotopologue ^16^O_3_ such vibrational state assigned as (701) in terms of normal modes has not yet been experimentally measured. Using the ab initio PES from [111], it was predicted [83] near 8422 cm^−1^, that is about 450 cm^−1^ higher in energy than for ^18^O_3_, while the (701) 27_720_ level of ^16^O_3_ would fall in the continuum above the first dissociation threshold. As the isotopic shifts between ^16^O_3_ and ^18^O_3_ are somewhat irregular [237], this means that the spectra of heavy isotopic species could bring new information about the levels near *D*_0_, which are not directly accessible by the bound-state transitions of the main isotopologue. This experimental information may also be important for the study of the deviations from the Born-Oppenheimer approximation [78,79,135].

#### 5.3.2. Exploring Large Amplitude Motions

Due to the Jahn-Teller effect [77,78,79], the ozone molecule has three identical C_2v_ potential wells in the ground electronic state separated by relatively high barriers between the equilibrium geometries [76,80]. This explains a somewhat surprising fact, confirmed experimentally, that a molecule consisting of three identical atoms behaves as an asymmetric top with three different rotational constants depending on vibrational excitation. “A permutation of the oxygen atoms makes the ozone molecule ‘travelling’ from one well to another” [33]. The interaction between nuclear motions in these three wells plays a crucial role in the isotopic exchange processes that exhibit anomalous properties [25,26,27,28,29,30,31].

Kokoouline et al. [32] have recently studied from the theoretical side the coupling between wavefunctions localized and delocalized in the three wells and their possible impacts on the vibrational band centers using the symmetrized ab initio “non-reef” PES of Tyuterev et al. [111]. The full symmetry approach described in [32,83] predicts that vibrational states should exhibit deviations from conventional spectroscopic models. According to calculations, the effect of this interaction critically depends on the type of the vibrational motion and was negligible for all vibrational states observed so far below 7920 cm^−1^. The transitions measured for the first time by Vasilchenko et al. [33] reach a vibrational state that is sensitive to this predicted effect [32]. Upper state assignments confirmed by the vibrational dependence of the rotational constants play a crucial role in the interpretation of the corresponding vibrational dynamics. The comparison of the available experimental data with ab initio calculations using a single-well approximation [111] and the three-wells approach [32] is discussed in the conclusion of [33].

The three lowest identical potential wells of C_2V_ symmetry (open ozone structure with an apex angle of about 117°) correspond to nuclear permutations. The ring minimum (D_3h_ symmetry) is located 0.3 eV above the O_2_ dissociation threshold and, therefore, does not matter for dynamic and spectroscopic applications [76,80,81]. A correct account of the interaction between the three identical potential wells is an important issue for the analyses of ozone spectra at high energy ranges. Recent theoretical results considering the three potential wells in a full symmetry approach predicted that “vibration states should exhibit deviations from the conventional one-potential-well ab initio calculations, near the dissociation threshold” [32].

A delocalization of wavefunctions [32] depends on the type of vibrational motion. Levels with high V_1_ quantum numbers of the symmetric normal modes q_1_ are not expected to be sensitive to this effect. Levels with big values of V_1_ up to 10 were experimentally accessed using dissociative resonance Raman spectroscopy [238], even though they correspond to energies much higher than the dissociation threshold D_0_. On the contrary, the vibrational motions corresponding to the simultaneous excitations of q_2_ and q_3_ modes at large energies at the flat range of the PES [32,111] could point towards another potential well. In this context, it was instructive to compare the observed band origins in the high energy range with ab initio predictions, using two types of recent variational calculations in a single well and in three potential wells but with the same ab initio surface [111]. The authors of publication [33] state: “we observe a better agreement of the experimentally determined energy value with the three-wells prediction for the ν_1_ + 6ν_2_ + 3ν_3_ band ^16^O_3_ involving large excitation of both bending (v_2_ = 6), and antisymmetric stretching v_3_ = 3. Despite the fact that it is still too early to claim for a complete experimental proof of the coupling effect, this is a hint confirming an interaction between the wells that needs further experimental confirmations”.

## 6. Discussions

In the past, there have been several reviews devoted to the analysis of high-resolution infrared spectra of ozone: Bacis and Flaud [85], Rinsland et al. [224], Campargue et al. [140], and Barbe et al. [93]. Each of them summarized the latest publications to date. Many corresponding results from various laboratories were incorporated in spectroscopic databanks, including dedicated line lists such as the atlas of ozone spectral transitions below 3000 cm^−1^ [87] and the “Spectroscopy and Molecular Properties of Ozone” (S&MPO) information system [18,21,64,239] as well as generic compilations GEISA [224] and HITRAN [24] for atmospheric applications. Some recent data are available via the VAMDC European web portal [240].

This review also updates the results in this domain and provides a lot of additional information, including a brief history of the discovery of ozone; issues of the formation and destruction of atmospheric ozone; a decrease of its concentration in the stratosphere and an increase in the troposphere; a possible solution of these problems and their potential connection with the supersonic air fleet; a brief overview of absorption in the UV, visible, and IR ranges; and the history of IR spectral recordings in various wavelength ranges and some other issues. 

First of all, it must be said that most of the new results presented in the review would not have been available without the development of experimental technologies that led to very sensitive CRDS setup [136,241,242,243] (and references therein). This revolutionary technology made it possible to register extremely weak ozone absorption spectra above 5800 cm^−1^, which would have been impossible using FTS. Thanks to these new spectral data, it becomes possible both to establish the limits of applicability of the method of effective Hamiltonians (the empirical approach) and to explore the validity of ab initio calculations.

In addition, from the point of view of experimental achievements, there are three main points. First, the issue of consistency between absolute intensities in the UV and IR spectral regions for the most absorbing intervals of 10 and 5 microns, which is critically important for the accuracy of the retrieval of atmospheric ozone concentrations, has been definitively resolved thanks to the recent works [22,23,63,173]. Second, high-resolution spectroscopy represents an excellent tool for checking the shapes of the dipole moment surfaces and the potential energy surfaces. Third, ro-vibrational patterns deduced from analyses of spectra can provide information for the improved modeling of the non-LTE effects in the upper atmosphere.

**Figure 20:**
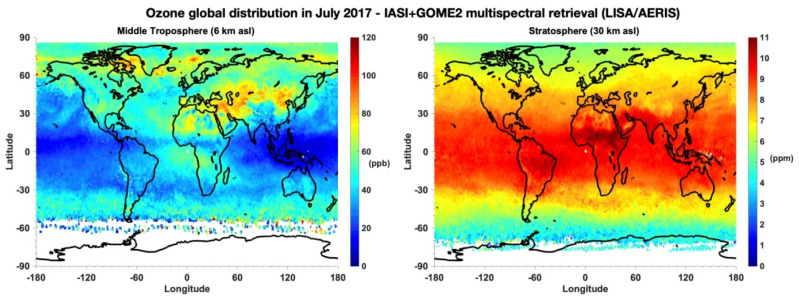
Illustration of the global distribution of ozone at the middle troposphere (left panel, 6 km of altitude above sea level—asl) and at the stratosphere (right panel, 30 km of altitude asl) averaged over the month of July 2017 retrieved with the IASI + GOME2 multispectral synergism of satellite measurements of IASI in the thermal infrared and GOME-2 in the ultraviolet (courtesy by J. Cuesta with permission) [244,245]. This satellite product has been developed by the LISA laboratory and it is publicly distributed and produced by the French data center AERIS (https://iasi.aeris-data.fr/o3_iago2/, provided on 29 December 2021) with support from the French Centre National des Etudes Spatiales (CNES).

Spectroscopy methods allow for an efficient analysis of the global distribution of ozone in Earth’s atmosphere using satellite measurements. As an example, satellite-derived maps of ozone distribution at the troposphere and stratosphere are illustrated in Figure 20. Tropospheric ozone enhancements associated with emissions of ozone precursors from anthropogenic activities and biomass burning is clearly seen over the Northern hemisphere (particularly over land) and also over Central-southern Africa. These satellite observations are derived from the multispectral synergism of IASI and GOME-2 measurements, respectively, in the thermal infrared and ultraviolet, which provides enhanced sensitivity to tropospheric ozone down to the lowermost troposphere (sensitivity peaking near 2 km of altitude over land and during summer, Cuesta et al., [244,245]).

The real-time evolution of global ozone distribution can be followed online at the dedicated web sites [1,2].

Concerning precise spectroscopic data, one can conclude, as stated in [17,18,21,159], that the two methods, the empirical approach and ab initio calculations, have complementary advantages with respect to the creation of the line lists which can be used for atmospheric applications. Ab initio methods allow for prediction of the centers and rotational constants for all vibrational bands in the ground electronic state with sufficient accuracy (in any case better than 1 cm^−1^) to explain a very large number of resonances (Coriolis or anharmonic), leading to significant perturbations in the positions of VR lines. Without these predictions, input information for the analysis of spectra in the high-energy range would be very limited. Using only an empirical approach it has been often impossible or, at best, incomplete and unreliable. However, ab initio calculations cannot nowadays achieve the experimental accuracy (about of 0.001 cm^−1^) for multi-electronic molecules that is currently obtained for the majority of lines using an empirical approach in the wavenumber range below 4000 cm^−1^.

As for the intensities, where the relative experimental accuracy is only about 1%, ab initio calculations play an essential role in ensuring a good consistency between various spectral regions [18,158]. They have brought an essential contribution [17,133] to the consistency between absolute intensities in 10 and 5 microns [20,22,23] at a sub-percent level. In addition, they make it possible to achieve higher intensity accuracy than direct observation of weak bands, for which very large values of (pressure × path length) are used and which are difficult to measure with sufficient precision. Finally, these calculations are able to predict all the hot bands [133], which are not directly observable, since most of them are hidden by stronger cold bands.

Recent validations of the ozone line lists carried out in the Jet Propulsion Laboratory, NASA by Toon [158] using atmospheric balloon, ground-based laboratory measurements simultaneously in 37 spectral infrared windows have indicated that band intensities corrected by ab initio calculation [18] and included in the last release of the S&MPO database were much more consistent from window to window than all other available lists in terms of variance-to-mean ratio scaling factors (VSF). The VSF dispersion in the range 630–4900 cm^−1^ were diminished by a factor of 2.7 when using new S&MPO_20d ozone line list with respect to HITRAN16 for the laboratory Kitt Peak spectra, by factor 1.9 for the balloon spectra, and by factor 2.1 for atmospheric ground-based observations. This permitted the reduction of the window-to-window intensity VSF root-means-squares deviations to 1.06%, 2.58% and 1.33%, correspondingly, for these types of experiments [158].

However, it should be noted that the variational calculations from ab initio surfaces are not accurate enough for the line positions and intensities in the case of accidental resonances and cannot provide sufficient accuracy, contrary to empirical works. From these remarks, it becomes obvious that the correct use of combined results can lead to a better agreement between calculated and experimental spectra, both laboratory and atmospheric.

Concerning the validation of ab initio potential functions by analyses of experimental spectra, it has been shown that the ro-vibrational levels and wavefunction are sensitive to the shape of the PES in the transition state range. Among all published ab initio surfaces of ozone, the NR_PES of [111], which does not exhibit a submerged barrier on the minimum energy path, has produced the best agreement with observed band origins and v-dependent rotational constants in the high energy range. This has also permitted a quantitative agreement in first-principle calculations of the rate constant for the O + O_2_ isotopic exchange reaction supported by the global ozone PES both in stationary [29,30] and time-dependent [31] approaches to be obtained.

On the other hand, recent observations [33,150] near the dissociation threshold can provide information on the density of the bound ro-vibrational state, whereas their analyses can give hints on a possible delocalization of wave-functions among potential wells [32].

Better knowledge of highly excited states could help improve the modeling of satellite observations of the emission/absorption of the IR radiation in non-LTE conditions of the upper atmosphere [246,247].

## Figures and Tables

**Table 1:** Room temperature absorption cross sections of ozone at 253.65 nm.

Value ^(^*^)^	Ref.
1147	[55]
1145	[56]
1157	[57]
1136	[58]
1143	[59]
1130.5	[60]
1123.9	[61]

^(^*^)^ in 10^−20^ cm^2^/molecule units.

**Table 2:** Mass, natural abundance, partition function, and statistical weigh of 18 possible ozone isotopologues.

Isotopic Species	Molar Mass (g/mol) [64]	Natural Abundance [64]	Partition Function [84]	Statistical Weight *g_i_* [84]
^16^O_3_	47.984745	0.992901	3475	1
^16^O^16^O^18^O	49.988991	3.98194 × 10^−3^	7584.6	1
^16^O^18^O^16^O	49.988991	1.99097 × 10^−3^	3703.0	1
^16^O^16^O^17^O	48.988960	7.40475 × 10^−4^	44044.0	6
^16^O^17^O^16^O	48.988960	3.70237 × 10^−4^	2147.2	6
^16^O^18^O^18^O	51.993237	8.384576 × 10^−6^	7937.6	1
^18^O^16^O^18^O	51.993237	4.192288 × 10^−6^	4070.3	1
^16^O^17^O^18^O	50.993206	1.554214 × 10^−6^	46563.0	6
^17^O^18^O^16^O	50.993206	1.554214 × 10^−6^	46074.0	6
^17^O^16^O^18^O	50.993206	1.554214 × 10^−6^	47135.0	6
^16^O^17^O^17^O	49.993179	2.88098 × 10^−7^	270516	36
^17^O^16^O^17^O	49.993179	1.44049 × 10^−7^	137040	1
^18^O_3_	53.997482	8.615 × 10^−9^	4264.7	1
^17^O^18^O^18^O	52.997452	3.194 × 10^−9^	49325	6
^18^O^17^O^18^O	52.997452	1.597 × 10^−9^	25005	6
^17^O^17^O^18^O	51.997421	5.92 × 10^−10^	281470	36
^17^O^18^O^17^O	51.997421	2.96 × 10^−10^	143420	1
^17^O_3_	50.997	5.5 × 10^−11^	841690	6

**Table 3:** Selection rules for IR dipole spectrum of ozone *.

Band Type	Electric Dipole Moment Component	Change in V_3_	Change in *K_a_*, *K_c_*
Symmetric species			
A	µ_z_ = µ_a_	e ↔ o	ee ↔ eo, oo ↔ oe
B	µ_x_ = µ_b_	e ↔ e o ↔ o	ee ↔ oo oe ↔ eo
Asymmetric species with hybrid bands			
A sub-band	µ_z_ = µ_a_	Any	ee ↔ eo, oo ↔ oe
B sub-band	µ_x_ = µ_b_	Any	ee ↔ oo, oe ↔ eo

* from [85] with permission by J.-M. Flaud.

**Table 4:** Nuclear spin degeneracy of VR levels for C_2V_ isotopic species containing two or three ^17^O atoms.

V	*K_a_*, *K_c_*	Degeneracy
e	ee, oo	15
	eo, oe	21
o	ee, oo	21
	eo, oe	15

**Table 5:** The list of the bands recorded using NSO Kitt Peak FTS in the years 1985 to 1988.

Bands	Spectral Range, cm^−1^	Ref.
ν_3_ − ν_2_	290–380	[177]
ν_3_	950–1250	[178,179,180,181]
ν_1_ + ν_2_, ν_2_ + ν_3_	1650–1900	[182,183,184]
2ν_3_, ν_1_ + ν_3_, 2ν_1_	1950–2210	[185,186]
2ν_3_ + ν_2_, ν_1_ + ν_2_ + ν_3_, 2ν_1_ + ν_2_	2600–2900	[187]
3ν_3_	2900–3206	[188]

**Table 6:** The list of the bands recorded using homemade GSMA FTS after 1993.

Bands	Spectral Range, cm^−1^	Ref.
ν_3_, ν_1_	900–1200	[21]
2ν_2_, 3ν_2_ − ν_2_	1300–1500	[189]
ν_1_ + ν_2_, ν_2_ + ν_3_	1550–1900	[21]
2ν_3_, ν_1_ + ν_3_, 2ν_1_	1900–2300	[21]
ν_1_ + 2ν_2_, 2ν_2_ + ν_3_	2400–2500	[190]
2ν_1_ + ν_2_, ν_1_ + ν_2_ + ν_3_, ν_2_ + 2ν_3_	2600–2900	[191]
ν_1_ + 3ν_2_, 3ν_2_ + ν_3_	3050–3200	[192]
3ν_1_	3250–3320	[193]
ν_1_ + 2ν_2_ + ν_3_, 2ν_2_ + 2ν_3_	3350–3480	[194]
ν_2_ + 3ν_3_, ν_1_ + ν_2_ + 2ν_3_	3650–3780	[195]
2ν_1_ + ν_2_ + ν_3_	3780–3880	[196]
4ν_3_, ν_1_ + 3ν_3_	3950–4050	[197]
3ν_1_ + 2ν_3_ − ν_3_	4070–4140	[159]
2ν_1_ + 2ν_3_	4100–4250	[198]
3ν_1_ + ν_3_	4200–4270	[199]
2ν_2_ + 3ν_3_	4280–4380	[200]
2ν_1_ + 2ν_2_ + ν_3_	4430–4530	[201]
ν_1_ + ν_2_ + 3ν_3_	4600–4680	[202]
2ν_1_ + ν_2_ + 2ν_3_	4740–4820	[203]
5ν_3_, 3ν_1_ + ν_2_ + ν_3_, ν_1_ + 4ν_3_	4850–4940	[204]
2ν_1_ + 3ν_3_	5020–5100	[205]
ν_1_ + 2ν_2_ + 3ν_3_, 4ν_1_ + ν_3_	5250–5350	[206]
2ν_1_ + ν_2_ + 3ν_3_	5480–5540	[207]
ν_1_ + ν_2_ + 4ν_3_, ν_2_ + 5ν_3_	5510–5730	[208]
ν_1_ + 5ν_3_	5750–5800	[209]

**Table 7:** Comparison of observed and calculated band centers for ^16^O_3_ in the spectral range above 5900 cm^−1^.

Band	Observation, cm^−1^	Ref.	Prediction_1 [132], cm^−1^	Δν_1 ^(^*^)^, cm^−1^	Prediction_2 [111], cm^−1^	Δν_2 ^(^*^)^, cm^−1^
ν_1_ + 3ν_2_ + 3ν_3_	5919.161	[136]	5919.040	0.121	5919.114	0.047
4ν_1_ + ν_2_ + ν_3_	5947.071	[136]	5947.107	−0.036	5946.296	0.775
3ν_2_ + 4ν_3_	6046.076	[137]	6046.020	0.056	6044.712	1.364
ν_1_ + 5ν_3_ II ^(^**^)^	6063.923	[137]	6063.642	0.281	6063.118	0.805
5ν_1_ + ν_2_	6100.216	[137]	6100.758	−0.542	6099.285	0.931
2ν_1_ + 2ν_2_ + 3ν_3_ I ^(^**^)^	6124.287	[137]	6124.557	−0.270	6124.998	−0.711
ν_1_ + 2ν_2_ + 4ν_3_ I ^(^**^)^	6154.702	[137]	6154.700	0.002	6155.528	−0.826
3ν_1_ + 3ν_2_ + ν_3_	6198.534	[137]	6198.234	0.300	6197.504	1.030
2ν_2_ + 5ν_3_	6305.047	[138]	6305.172	−0.125	6303.590	1.457
ν_1_ + 2ν_2_ + 4ν_3_ II ^(^**^)^	6343.983	[138]	6343.616	0.367	6343.946	0.037
5ν_1_ + ν_3_	6355.722	[138]	6355.774	−0.052	6354.762	0.960
4ν_1_ + 3ν_2_	6365.264	[138]	6365.588	−0.324	6365.961	−0.697
2ν_1_ + 2ν_2_ + 3ν_3_ II ^(^**^)^	6386.997	[138]	6386.795	0.202	6388.120	−1.123
4ν_2_ + 4ν_3_	6506.129	[141]	6505.850	0.279	6505.560	0.569
4ν_1_ + 2ν_2_ + ν_3_	6567.841	[139]	6567.898	−0.057	6567.978	−0.137
2ν_1_ + 5ν_3_ I ^(^**^)^	6586.967	[139]	6586.867	0.100	6588.945	−1.978
2ν_1_ + 3ν_2_ + 3ν_3_ I ^(^**^)^	6716.536	[140]	6716.676	−0.140	6717.469	−0.933
5ν_1_ + 2ν_2_	6750.908	[140]	6751.218	−0.310	6749.626	1.282
2ν_1_ + 4ν_2_ + 2ν_3_	6764.789	[140]	6764.594	0.195	6765.486	−0.697
3ν_2_ + 5ν_3_	6895.487	[139]	6895.463	0.024	6895.133	0.354
ν_1_ + 6ν_3_	6928.836	[139]	6929.149	−0.313	6929.393	−0.557
5ν_1_ + ν_2_ + ν_3_	6981.870	[139]	6982.174	−0.304	6980.684	1.186
2ν_1_ + 3ν_2_ + 3ν_3_ II ^(^**^)^	6990.069	[139]	6989.615	0.454	6990.224	−0.155
ν_1_ + 2ν_2_ + 5ν_3_	7130.774	[141]	7130.307	0.467	7133.306	−2.532
ν_1_ + 5ν_2_ + 3ν_3_	7286.774	[141]	7286.239	0.535	7288.176	−1.402
6ν_1_ + ν_3_	7394.801	[142]	7395.350	−0.549	7393.579	1.222
3ν_1_ + 5ν_2_ + ν_3_	7446.067	[142]	7445.333	0.734	7445.558	0.509
2ν_1_ + 4ν_2_ + 3ν_3_	7452.323	[142]	7450.753	1.570	7452.810	−0.487
5ν_1_ + 2ν_2_ + ν_3_	7578.828	[142]	7577.549	1.279	7577.974	0.854
2ν_1_ + 4ν_2_ + 3ν_3_	7686.081	[143]	7684.854	1.227	7687.224	−1.143
2ν_1_ + 7ν_2_ + ν_3_	7739.606	[143]	7737.555	2.051	7741.147	−1.541
4ν_1_ + 5ν_3_	7860.077	[143]	7857.863	2.214	7863.318	−3.241
6ν_1_ + ν_2_ + ν_3_	7992.831	[33]	7993.360	−0.529	7991.660	1.171
			*RMS*	0.72		1.17

^(^*^)^ Δν = ν^OBS^ − ν^PRED^, ^(^**^)^ According to the principal terms in the normal mode decomposition of the upper state wavefunction, the levels appear twice as majoritary.

**Table 8:** Comparison of experimental and predicted rotational constants of ^16^O_3_ for the bands above 5900 cm^−1^.

Band	Ref	*A* ^OBS^	Δ*A* ^(^*^)^, (%)	*B* ^OBS^	Δ*B* ^(^*^)^, (%)	*C* ^OBS^	Δ*C* ^(^*^)^, (%)
ν_1_ + 3ν_2_ + 3ν_3_	[136]	3.5942	0.30	0.4242	0.66	0.3695	0.68
4ν_1_ + ν_2_ + ν_3_	[136]	3.5089	0.25	0.4271	0.35	0.3768	0.48
3ν_2_ + 4ν_3_	[137]	3.5716	0.43	0.4239	0.47	0.3710	0.62
ν_1_ + 5ν_3_ II ^(^**^)^	[137]	3.3843	0.31	0.4234	0.40	0.3721	0.59
5ν_1_ + ν_2_	[137]	3.5949	0.04	0.4306	0.42	0.3927	0.23
2ν_1_ + 2ν_2_ + 3ν_3_ I ^(^**^)^	[137]	3.4737	0.28	0.4197	0.48	0.3671	0.60
ν_1_ + 2ν_2_ + 4ν_3_ I ^(^**^)^	[137]	3.5184	0.34	0.4217	0.57	0.3675	0.44
3ν_1_ + 3ν_2_ + ν_3_	[137]	3.5956	0.19	0.4257	0.40	0.3732	0.32
2ν_2_ + 5ν_3_	[138]	3.4263	0.24	0.4201	0.41	0.3656	0.55
ν_1_ + 2ν_2_ + 4ν_3_ II ^(^**^)^	[138]	3.4424	−0.87	0.4174	−0.36	0.3690	−0.03
5ν_1_ + ν_3_	[138]	3.4701	0.28	0.4267	0.28	0.3773	0.27
4ν_1_ + 3ν_2_	[138]	3.6094	0.58	0.4263	0.54	0.3803	0.61
2ν_1_ + 2ν_2_ + 3ν_3_ II ^(^**^)^	[138]	3.4551	0.33	0.4204	0.43	0.3683	0.46
4ν_2_ + 4ν_3_	[141]	3.6030	0.25	0.4199	0.41	0.3643	0.36
4ν_1_ + 2ν_2_ + ν_3_	[139]	3.4387	0.13	0.4200	0.79	0.3691	0.60
2ν_1_ + 5ν_3_ I ^(^**^)^	[139]	3.4237	0.36	0.4194	0.33	0.3696	0.38
2ν_1_ + 3ν_2_ + 3ν_3_ I ^(^**^)^	[140]	3.5135	−0.07	0.4158	0.36	0.3625	0.44
5ν_1_ + 2ν_2_	[140]	3.5905	−0.39	0.4278	0.47	0.3858	0.16
2ν_1_ + 4ν_2_ + 2ν_3_	[140]	3.5938	0.09	0.4198	0.55	0.3661	0.55
3ν_2_ + 5ν_3_	[139]	3.4376	−0.22	0.4165	0.41	0.3672	1.72
ν_1_ + 6ν_3_	[139]	3.4076	1.05	0.4179	0.38	0.3670	0.58
5ν_1_ + ν_2_ + ν_3_	[139]	3.4790	0.25	0.4234	0.31	0.3738	0.35
2ν_1_ + 3ν_2_ + 3ν_3_ II ^(^**^)^	[139]	3.5343	0.32	0.4191	0.26	0.3579	−0.86
ν_1_ + 2ν_2_ + 5ν_3_	[141]	3.3863	0.33	0.4124	0.56	0.3619	0.67
ν_1_ + 5ν_2_ + 3ν_3_	[141]	3.5439	0.08	0.4116	1.31	0.3579	−0.86
6ν_1_ + ν_3_	[142]	3.4592	0.23	0.4237	0.33	0.3743	0.21
3ν_1_ + 5ν_2_ + ν_3_	[142]	3.6129	−0.22	0.4155	0.05	0.3618	−0.03
2ν_1_ + 4ν_2_ + 3ν_3_	[142]	3.4877	1.14	0.4116	0.66	0.3569	0.51
5ν_1_ + 2ν_2_ + ν_3_	[142]	3.4936	0.18	0.4141	0.49	0.3594	0.48
2ν_1_ + 4ν_2_ + 3ν_3_	[143]	3.4471	−0.10	0.4136	0.61	0.3611	0.50
2ν_1_ + 7ν_2_ + ν_3_	[143]	3.5578	−0.04	0.4058	0.40	0.3514	0.00
4ν_1_ + 5ν_3_	[143]	3.4604	0.70	0.4087	0.27	0.3583	0.08
ν_1_ + 6ν_2_ + 3ν_3_	[33]	3.4484	−0.67	0.4018	1.59	0.3409	−1.90
6ν_1_ + ν_2_ + ν_3_	[33]	3.4136	0.19	0.4172	0.41	0.3687	0.66

^(^*^)^ Relative difference (Δ*P*) for all three rotational constants (*P* = *A*, *B* and *C*) is $\Delta P\;=\;\frac{P^{OBS}-\;P^{PRED}}{P^{PRED}}\;\times \;100\%$. Predictions for the v-dependent rotational constants were carried out using the PES of [132]. ^(^**^)^ According to the principal terms in the normal mode decomposition of the upper state wavefunction, the levels appear twice as majoritary.

**Table 9:** Comparison of observed and calculated band centers for ^18^O_3_ in the spectral range above 5900 cm^−1^.

Band	Observation, cm^−1^	Ref.	Prediction_1 [132], cm^−1^	Δν_1 ^(^*^)^, cm^−1^	Prediction_2 [111], cm^−1^	Δν_2 ^(^*^)^, cm^−1^
2ν_2_ + 5ν_3_	5984.439	[144]	5984.638	−0.199	5982.657	1.782
4ν_1_ + 3ν_2_	6011.836	[144]	6012.226	−0.390	6011.488	0.348
5ν_1_ + ν_3_	6013.048	[144]	6013.140	−0.092	6012.227	0.821
2ν_1_ + ν_2_ + 4ν_3_	6047.101	[144]	6047.406	−0.305	6048.271	−1.170
ν_1_ + ν_2_ + 5ν_3_	6072.132	[144]	6072.222	−0.090	6073.575	−1.443
ν_2_ + 6ν_3_	6245.001	[145]	6245.323	−0.322	6244.102	0.899
2ν_1_ + 5ν_3_	6270.604	[145]	6270.754	−0.150	6273.004	−2.400
3ν_1_ + 4ν_3_	6296.358	[145]	6296.167	0.191	6297.401	−1.043
2ν_1_ + 3ν_2_ + 3ν_3_ I ^(^**^)^	6392.214	[145]	6392.621	−0.407	6392.891	−0.677
3ν_2_ + 5ν_3_	6556.786	[146]	6557.018	−0.232	6555.813	0.973
ν_1_ + 3ν_2_ + 4ν_3_	6592.066	[146]	6593.006	−0.940	6593.628	−1.562
5ν_1_ + ν_2_ + ν_3_	6611.039	[146]	6611.306	−0.267	6610.079	0.960
2ν_1_ + 3ν_2_ + 3ν_3_ II ^(^**^)^	6642.897	[146]	6642.901	−0.004	6643.808	−0.911
ν_1_ + 2ν_2_ + 5ν_3_	6796.461	[146]	6796.642	−0.181	6797.736	−1.275
4ν_1_ + 3ν_2_ + ν_3_	6825.512	[146]	6825.632	−0.120	6826.395	−0.883
3ν_1_ + 5ν_3_	7009.087	[147]	7009.059	0.028	7012.075	−2.988
ν_2_ + 7ν_3_	7101.476	[147]	7101.297	0.179	7101.159	0.317
4ν_2_ + 5ν_3_	7115.370	[147]	7115.334	0.036	7115.051	0.319
ν_1_ + 6ν_2_ + 3ν_3_I ^(^**^)^	7487.140	[148]	7486.308	0.832	7488.614	−1.474
ν_1_ + 3ν_2_ + 5ν_3_	7503.393	[148]	7502.645	0.748	7505.893	−2.500
4ν_1_ + 4ν_2_ + ν_3_	7528.519	[148]	7527.863	0.656	7529.154	−0.635
ν_1_ + 6ν_2_ + 3ν_3_II ^(^**^)^	7629.536	[148]	7627.934	1.602	7632.176	−2.640
3ν_1_ + 6ν_2_ + ν_3_	7752.699	[148]	7751.420	1.279	7752.035	0.664
2ν_1_ + 8ν_2_ + ν_3_	7908.840	[148]	7906.350	2.490	7910.411	−1.571
7ν_1_ + ν_3_	7974.022	[149]	7975.349	−1.327	7972.771	1.251

See footnotes of Table 7.

**Table 10:** Comparison of the experimental and predicted rotational constants of ^18^O_3_ for the bands above 5900 cm^−1^.

Band	Ref	*A* ^OBS^	Δ*A* ^(^*^)^, (%)	*B* ^OBS^	Δ*B* ^(^*^)^, (%)	*C* ^OBS^	Δ*C* ^(^*^)^, (%)
2ν_2_ + 5ν_3_	[144]	3.0646	0.28	0.3758	0.40	0.3275	0.74
4ν_1_ + 3ν_2_	[144]	3.2504	0.09	0.3828	0.76	0.3419	0.53
5ν_1_ + ν_3_	[144]	3.0907	0.30	0.3805	0.50	0.3367	0.27
2ν_1_ + ν_2_ + 4ν_3_	[144]	3.0557	0.22	0.3739	0.37	0.3284	0.09
ν_1_ + ν_2_ + 5ν_3_	[144]	3.0559	0.25	0.3745	0.46	0.3284	0.37
ν_2_ + 6ν_3_	[145]	2.9517	−0.34	0.3727	0.35	0.3263	0.58
2ν_1_ + 5ν_3_	[145]	2.9872	0.36	0.3721	0.40	0.3275	0.52
3ν_1_ + 4ν_3_	[145]	3.0039	0.18	0.3740	0.40	0.3282	0.77
2ν_1_ + 3ν_2_ + 3ν_3_I ^(^**^)^	[145]	3.1256	0.21	0.3721	0.27	0.3243	0.18
3ν_2_ + 5ν_3_	[146]	3.0893	0.26	0.3725	0.38	0.3241	0.50
ν_1_ + 3ν_2_ + 4ν_3_	[146]	3.0776	−0.16	0.3708	0.32	0.3244	0.24
5ν_1_ + ν_2_ + ν_3_	[146]	3.1059	0.21	0.3782	0.48	0.3347	0.51
2ν_1_ + 3ν_2_ + 3ν_3_II ^(^**^)^	[146]	3.1239	0.11	0.3731	0.32	0.3276	0.40
ν_1_ + 2ν_2_ + 5ν_3_	[146]	3.0369	0.12	0.3699	0.38	0.3238	0.03
4ν_1_ + 3ν_2_ + ν_3_	[146]	3.1282	0.26	0.3738	0.46	0.3282	0.58
3ν_1_ + 5ν_3_	[147]	2.9578	0.22	0.3682	0.32	0.3241	0.53
ν_1_ + 7ν_3_	[147]	3.0315	0.45	0.3696	0.41	0.3221	0.50
4ν_2_ + 5ν_3_	[147]	3.0722	0.16	0.3695	0.32	0.3224	0.46
ν_1_ + 6ν_2_ + 3ν_3_ I ^(^**^)^	[148]	3.1866	0.19	0.3644	0.27	0.3169	0.54
ν_1_ + 3ν_2_ + 5ν_3_	[148]	3.0632	0.11	0.3665	0.19	0.3215	0.53
4ν_1_ + 4ν_2_ + ν_3_	[148]	3.1369	0.65	0.3711	0.49	0.3243	0.31
ν_1_ + 6ν_2_ + 3ν_3_ II ^(^**^)^	[148]	3.0966	0.09	0.3606	0.25	0.3135	0.00
3ν_1_ + 6ν_2_ + ν_3_	[148]	3.0984	0.89	0.3673	0.14	0.3190	0.56
2ν_1_ + 8ν_2_ + ν_3_	[148]	3.1400	0.20	0.3580	0.25	0.3096	0.81
7ν_1_ + ν_3_	[149]	3.0780	0.25	0.3755	0.37	0.3319	0.35

See footnotes of Table 8.

**Table 11:** Comparison of observed and calculated band centers for the ^16^O^16^O^18^O, ^16^O^18^O^16^O, ^16^O^18^O^18^O, and ^18^O^16^O^18^O species in the spectral range above 5900 cm^−1^.

Band	Observation, cm^−1^	Ref.	Prediction_1 [132], cm^−1^	Δν_1 ^(^*^)^, cm^−1^
^16^O^16^O^18^O				
2ν_1_ + 2ν_2_ + 3ν_3_ I ^(^**^)^	6026.084	[150]	6026.13	−0.05
2ν_2_ + 5ν_3_	6213.492	[150]	6213.56	−0.07
5ν_1_ + ν_3_	6276.706	[150]	6276.71	0.00
2ν_1_ + 2ν_2_ + 3ν_3_ II ^(^**^)^	6325.213	[150]	6325.38	−0.17
^16^O^18^O^16^O				
2ν_1_ + 2ν_2_ + 3ν_3_ I ^(^**^)^	5983.636	[151]	5983.98	−0.35
2ν_2_ + 5ν_3_	6151.385	[151]	6151.66	−0.27
5ν_1_ + ν_3_	6182.300	[151]	6182.51	−0.21
2ν_1_ + 2ν_2_ + 3ν_3_ II ^(^**^)^	6225.299	[151]	6225.19	0.06
^16^O^18^O^18^O				
2ν_2_ + 5ν_3_	6054.647	[152]	6055.08	−0.43
2ν_1_ + 2ν_2_ + 3ν_3_ II ^(^**^)^	6168.355	[152]	6168.76	−0.40
^18^O^16^O^18^O				
2ν_1_ + 2ν_2_ + 3ν_3_ I ^(^**^)^	5964.749	[152]	5964.97	−0.23
5ν_1_ + ν_3_	6195.364	[152]	6195.28	0.08
3ν_1_ + ν_2_ + 3ν_3_	6240.484	[152]	6240.43	0.06
2ν_1_ + 5ν_3_	6457.448	[153]	6457.45	0.10
ν_1_ + 4ν_2_ + 3ν_3_	6535.823	[153]	6536.07	−0.25
3ν_2_ + 5ν_3_	6713.422	[153]	6713.43	−0.01

See footnotes of Table 7.

**Table 12:** Comparison of observed and predicted rotational constants of the ^16^O^16^O^18^O, ^16^O^18^O^16^O, ^16^O^18^O^18^O, and ^18^O^16^O^18^O species for the bands above 5900 cm^−1^.

Band	Ref.	*A* ^OBS^	Δ*A* ^(^*^)^, (%)	*B* ^OBS^	Δ*B* ^(^*^)^, (%)	*C* ^OBS^	Δ*C* ^(^*^)^, (%)
^16^O^16^O^18^O							
2ν_1_ + 2ν_2_ + 3ν_3_ I ^(^**^)^	[150]	3.4472	−0.09	0.3974	−0.50	0.3487	0.37
2ν_2_ + 5ν_3_	[150]	3.3752	−0.03	0.3962	−0.32	0.3466	0.20
5ν_1_ + ν_3_	[150]	3.4137	−0.07	0.4028	−0.32	0.3575	0.44
2ν_1_ + 2ν_2_ + 3ν_3_ II ^(^**^)^	[150]	3.4000	−0.18	0.3972	−0.05	0.3504	0.00
^16^O^18^O^16^O							
2ν_1_ + 2ν_2_ + 3ν_3_I ^(^**^)^	[151]	3.2182	0.31	0.4208	0.14	0.3652	0.16
2ν_2_ + 5ν_3_	[151]	3.1852	0.30	0.4212	−0.19	0.3651	−0.21
5ν_1_ + ν_3_	[151]	3.2045	0.30	0.4272	0.35	0.3759	0.16
2ν_1_ + 2ν_2_ + 3ν_3_ II ^(^**^)^	[151]	3.200	0.26	0.4216	0.19	0.3672	−0.98
^16^O^18^O^18^O							
2ν_2_ + 5ν_3_	[152]	3.1214	0.25	0.3968	0.45	0.3454	0.58
2ν_1_ + 2ν_2_ + 3ν_3_ II ^(^**^)^	[152]	3.1412	0.26	0.3980	0.40	0.3486	0.17
^18^O^16^O^18^O							
2ν_1_ + 2ν_2_ + 3ν_3_ I ^(^**^)^	[152]	3.3638	0.29	0.3746	0.07	0.3302	0.15
5ν_1_ + ν_3_	[152]	3.3566	0.26	0.3788	0.34	0.3371	0.21
3ν_1_ + ν_2_ + 3ν_3_	[152]	3.3133	0.54	0.3736	−0.80	0.3296	1.09
2ν_1_ + 5ν_3_	[153]	3.2315	0.32	0.3709	0.40	0.3282	0.66
ν_1_ + 4ν_2_ + 3ν_3_	[153]	3.3786	0.25	0.3715	0.40	0.3264	0.48
3ν_2_ + 5ν_3_	[153]	3.3288	0.25	0.3711	0.45	0.3249	0.18

See footnotes of Table 8.

**Table 13:** Comparison of *RMS*, mean, and integrated deviations for line intensities between the S&MPO_20d line list and experimental values of LERMA/MONARIS in the 10 and 5 microns range for ^16^O_3_ as reported in [24].

Range	Bands	*N ^*(*a*)*^*	Δ(*S*_v_) ^(*b*)^	*RMS* (*S*)	Mean (*S*)
10 μm	ν_3_, ν_1_, ν_2_ +ν_3_ − ν_2_	497	+0.28%	0.78%	+0.26%
5 μm	ν_1_ + ν_3_, 2ν_3_	319	−0.04%	0.37%	−0.02%

*^*(*a*)*^ N* is the number of the line intensities included for the comparison, and *S*_v_ is the sum of intensities.*^*(*b*)*^* Relative deviations of sums of all line intensities.

**Table 14:** Isotopic abundance of two gas mixtures used for the spectrum registrations.

Species	Pressure (in Torr)	
	Mixture 1	Mixture 2
^16^O^16^O^16^O	0.0085	0.205
^16^O^16^O^18^O	0.0094	0.322
^16^O^18^O^16^O	0.0047	0.191
^16^O^16^O^17^O	0.0062	0.315
^16^O^17^O^16^O	0.0031	0.134
^16^O^18^O^18^O	0.0052	0.390
^18^O^16^O^18^O	0.0026	0.141
^17^O^16^O^18^O	0.0034	0.187
^16^O^18^O^17^O	0.0034	0.209
^16^O^17^O^18^O	0.0034	0.192
^16^O^17^O^17^O	0.2277	0.198
^17^O^16^O^17^O	0.1139	0.086
^18^O^18^O^18^O	0.0014	0.106
^17^O^18^O^18^O	0.0019	0.165
^18^O^17^O^18^O	0.0096	0.088
^17^O^17^O^18^O	0.1258	0.172
^17^O^18^O^17^O	0.0629	0.063
^17^O^17^O^17^O	0.4163	0.050

**Table 15:** Summary of experimental results and analyses for the ν_3_ bands of eighteen isotopic species.

Species	*J_max_*	*K_a max_*	*NT*	*RMS*, cm^−1^	Ref.	Comment
^16^O^16^O^16^O	81	23	3741	0.12	[180]	TW
^16^O^16^O^18^O	57	15	1455	0.57	[228]	TW
^16^O^18^O^16^O	52	17	489	0.56	[229]	
^16^O^16^O^17^O	54	11	466	0.63	[230]	
^16^O^17^O^16^O	59	9	413	0.45	[231]	
^16^O^18^O^18^O	61	14	1319	0.26	[232]	TW
^18^O^16^O^18^O	60	15	762	0.57	[233]	
^16^O^17^O^18^O	***52***	***12***	***2377***	***0.47***		unpublished
^17^O^18^O^16^O	***58***	***11***	***1764***	***0.44***		unpublished
^17^O^16^O^18^O	***52***	***10***	***1535***	***0.48***		unpublished
^16^O^17^O^17^O	52	11	1378	0.68	[234]	TW
^17^O^16^O^17^O	50	12	1091	0.43	[235]	TW
^18^O^18^O^18^O	61	14	1319	0.26	[233]	TW
^17^O^18^O^18^O	***70***	***13***	***2373***	***0.49***		unpublished
^18^O^17^O^18^O	***57***	***11***	***1391***	***0.31***		unpublished
^17^O^17^O^18^O	***46***	***12***	***2304***	***0.49***		unpublished
^17^O^18^O^17^O	***52***	***11***	***1042***	***0.64***		unpublished
^17^O^17^O^17^O	59	12	1283	0.49	[236]	TW

**Table 16:** Comparison between empirical and calculated ν_3_ band centers.

Species	ν^OBS^, cm^−1^	ν^CALC^, [132] cm^−1^	*d*ν, cm^−1^
^16^O^16^O^16^O	1042.0839	1041.888	0.196
^16^O^16^O^18^O	1028.1120	1027.918	0.194
^16^O^18^O^16^O	1008.4530	1008.268	0.185
^16^O^16^O^17^O	1035.3590	1035.173	0.186
^16^O^17^O^16^O	1024.3955	1024.213	0.182
^16^O^18^O^18^O	993.9257	993.753	0.173
^18^O^16^O^18^O	1019.3498	1019.163	0.187
^16^O^17^O^18^O	1010.1334	1009.950	0.183
^17^O^18^O^16^O	1024.1008	1023.914	0.187
^17^O^16^O^18^O	1001.4682	1001.285	0.183
^16^O^17^O^17^O	1017.5340	1017.348	0.186
^17^O^16^O^17^O	1030.0946	1029.907	0.188
^18^O^18^O^18^O	984.8187	984.647	0.172
^17^O^18^O^18^O	989.7595	989.583	0.176
^18^O^17^O^18^O	1001.1958	1001.019	0.177
^17^O^17^O^18^O	1006.0435	1005.863	0.180
^17^O^18^O^17^O	995.9944	995.817	0.177
^17^O^17^O^17^O	1012.1613	1011.979	0.182

**Table 17:** Comparison between empirical and predicted ^(^*^)^ rotational constants *A*, *B*, and *C* for the (001) vibration state.

Species	*A^OBS^*	*A^CALC^*	ν*A* (%)	*B^OBS^*	*B^CALC^*	ν*B* (%)	*C^OBS^*	*C^CALC^*	ν*C* (%)
^16^O^16^O^16^O	3.5000	3.4893	0.30	0.4413	0.440	0.29	0.3909	0.3862	1.23
^16^O^16^O^18^O	3.4396	3.4278	0.35	0.4160	0.4142	0.43	0.367	0.3649	0.57
^16^O^18^O^16^O	3.2435	3.2320	0.35	0.4415	0.4394	0.47	0.3882	0.3822	1.55
^16^O^16^O^17^O	3.4665	3.4554	0.32	0.4281	0.4261	0.47	0.3804	0.3744	1.57
^16^O^17^O^16^O	3.3637	3.3527	0.33	0.4415	0.4395	0.46	0.3899	0.3847	1.35
^16^O^18^O^18^O	3.1822	3.1709	0.36	0.4165	0.4146	0.46	0.3640	0.3620	0.54
^18^O^16^O^18^O	3.3711	3.3594	0.35	0.3923	0.3905	0.46	0.3509	0.3447	1.82
^16^O^17^O^18^O	3.3032	3.2914	0.36	0.4164	0.4142	0.53	0.3694	0.3639	1.52
^17^O^18^O^16^O	3.4026	3.3905	0.33	0.4037	0.4017	0.50	0.3599	0.3544	1.55
^17^O^16^O^18^O	3.2102	3.1991	0.34	0.4286	0.4268	0.42	0.3775	0.3725	1.34
^16^O^17^O^17^O	3.3303	3.3197	0.32	0.4283	0.4266	0.39	0.3789	0.3740	1.31
^17^O^16^O^17^O	3.4317	3.4200	0.34	0.4153	0.4134	0.46	0.3696	0.3639	1.58
^18^O^18^O^18^O	3.1139	3.1123	0.05	0.3926	0.3929	−0.07	0.3482	0.3530	−1.33
^17^O^18^O^18^O	3.1455	3.1339	0.35	0.4050	0.4020	0.75	0.3577	0.3525	1.34
^18^O^17^O^18^O	3.2374	3.2233	0.36	0.4258	0.4268	0.42	0.3775	0.3725	1.34
^17^O^17^O^18^O	3.2661	3.2547	0.35	0.4038	0.4022	0.39	0.3597	0.3542	1.35
^17^O^18^O^17^O	3.1748	3.1635	0.36	0.4156	0.4136	0.48	0.3665	0.3618	1.30
^17^O^17^O^17^O	3.2955	3.2840	0.35	0.4154	0.4135	0.46	0.3682	0.3631	1.38

^(^*^)^ Predictions for the v-dependent rotational constants were carried out using the PES of [132].

## Data Availability

Not applicable.

## Sample Availability

Samples of the compounds are not available from the authors.
